# 2,6-Bis((benzoyl-R)amino)pyridine (R = H, 4-Me, and 4-NMe_2_) Derivatives for the Removal of Cu(II), Ni(II), Co(II), and Zn(II) Ions from Aqueous Solutions in Classic Solvent Extraction and a Membrane Extraction

**DOI:** 10.3390/membranes11040233

**Published:** 2021-03-25

**Authors:** Daria Bożejewicz, Borys Ośmiałowski, Małgorzata Anna Kaczorowska, Katarzyna Witt

**Affiliations:** 1 Faculty of Chemical Technology and Engineering, UTP University of Science and Technology, 3 Seminaryjna, PL 85326 Bydgoszcz, Poland; malgorzata.kaczorowska@utp.edu.pl (M.A.K.); katarzyna.witt@utp.edu.pl (K.W.); 2 Faculty of Chemistry, Nicolaus Copernicus University, 7 Gagarin Street, PL 87100 Toruń, Poland; borys.osmialowski@umk.pl

**Keywords:** 2,6-bis((benzoyl-R)amino)pyridine derivatives, metal ions Cu(II), Ni(II), Co(II), Zn(II), membranes, solvent extraction, mass spectrometry, nuclear magnetic resonance

## Abstract

In this paper, the application of new substituted 2,6-bis((benzoyl-R)amino)pyridine (R = H, 4-Me, and 4-NMe_2_) derivatives for the recovery of copper(II), nickel(II), cobalt(II), and zinc(II) ions from aqueous solutions was described. The structures of the synthesized compounds were confirmed by nuclear magnetic resonance spectroscopy (NMR), electrospray ionization high-resolution mass spectrometry (ESI HRMS), and tandem mass spectrometry methods (HCD MS/MS). Three different derivatives of 2,6-bis((benzoyl-R)amino)pyridine were used as carriers in membrane processes and as extractants in classic solvent extraction. In each case, the single derivative recovery was carried out on a model solution that contained only one type of metal ions. Spectrophotometry studies were performed to determine the stability constants of the complexes formed by the synthesized species with analyzed metals ions. The results obtained indicate that the synthesized compounds form stable complexes with Cu(II), Ni(II), Co(II), and Zn(II) ions and can be used in both types of studied recovery processes. However, the effectiveness of the synthesized compounds in the recovery of metal ions depends both on the structure of compounds and properties of metals as well as on their concentration.

## 1. Introduction

Schiff bases, also referred to as imines, are chemical compounds containing azomethine functional group (–R^1^HC=NR^2^, where R^1^, R^2^—hydrogen atom, alkyl, or aryl), and are usually formed by the condensation reaction of a primary amine with an active carbonyl compound (aldehyde or ketone) [1,2]. Schiff bases are a nitrogen analogue of an aldehyde or ketone in which the carbonyl group (C=O) is replaced by an imine (C=N) [3]. Imines have very good donor properties and can form chemically stable complex compounds with various metals [4,5,6] including transient metal ions [1,7], which makes them useful and widely used in coordination chemistry as organic ligands [8,9].

Many Schiff bases have been successfully used in separation processes for the recovery of metal ions (e.g., N,N′-bis(salicylidene)ethylenediamine (Salen) was applied for the removal of copper(II) ions by solvent extraction [10]) and also for the recovery of copper(II), nickel(II), and zinc(II) ions by liquid–liquid extraction and by using polymer inclusion membrane sorption/desorption and transport across the membrane [11]. Salen derivatives with different electron-accepting substituents on the aromatic ring have been utilized as extractant agents in polymeric inclusion membranes for the extraction of gold ions from aqueous solutions [12]. Other Schiff bases have also been utilized as metal ion extractants. Oshima et al. used the following imines for the selective removal of copper(II) ions: N,N′-bis(2-quinolylmethylidene)-1,2-diiminoethane (BQIE), N,N′-bis(2-pyridylmethylidene)-1,3-diimino-2,2-dimethylpropane (BPMP), and N,N′-bis(2-quinolylmethylidene)-1,3-diimino-2,2-dimethylpropane (BQMP). They found that the distance between two imine N atoms in these complexes was a factor controlling the extraction selectivity process [13]. Dede et. al. used 1-(biphenyl)-2-hydroxyimino-2-(4-chloroanilino)-1-ethanone, 1-(biphenyl)-2-hydroxyimino-2-(4-methylanilino)-1-ethanone, and 1-(biphenyl)-2-hydroxyimino-2-(N-pyrrolidino)-1-ethanone for the removal of transition metal ions such as Mn(II), Co(II), Ni(II), Cu(II), Zn(II), Pb(II), Cd(II), and Hg(II) through a solvent extraction process [14]. Recently, Wiecka and coworkers applied pyridinium derivatives containing an imidoamide or imine moiety as novel extractants for the recovery of palladium(II) and platinum(IV) from model chloride aqueous solutions [15], while Bhargava and Uma synthesized a new Schiff base called N-(4-hydroxy-3-methoxy benzylidene)-biphenyl-4-amine) and used it for the adsorption of copper(II) ions [16]. Another compound, (E)-4-(2-hydroxyethyl imino)pentan-2-one (AcEt), was utilized during solvent extraction in the separation of thorium(IV) metal ions from chloride media [17]. Schiff bases were also applied in polymer membranes for the recovery of metal ions (e.g., Mashhadizadeh and Sheikhshoaie used a bis[5-((4-nitrophenyl)azo salicylaldehyde as a carrier in polymer inclusion membrane for the removal of mercury(II) ions [18]). Other carrier groups applicable to the removal of metal ions are calixarenes (e.g., Ulewicz and co-authors used a p-tert-butylcalix[4]arene derivative to transport Pb(II) ions across PIMs [19], whereas a calix[4]-crown-6-derivative nest carrier was used to recover Zn(II), Cd(II), and Pb(II) ions by a PIM membrane [20]). However, research on the recovery of different metal ions from aqueous solutions is not based solely on the search for new compounds (extractants and carriers); well-known compounds with relatively simple structures are increasingly being used for this purpose [21,22]. Lately, 2,6-diaminopyridine has been applied for the removal of copper(II) and zinc(II) ions from aqueous solutions in a polymer inclusion membrane and in a classic solvent extraction processes [23].

**Figure 1:**
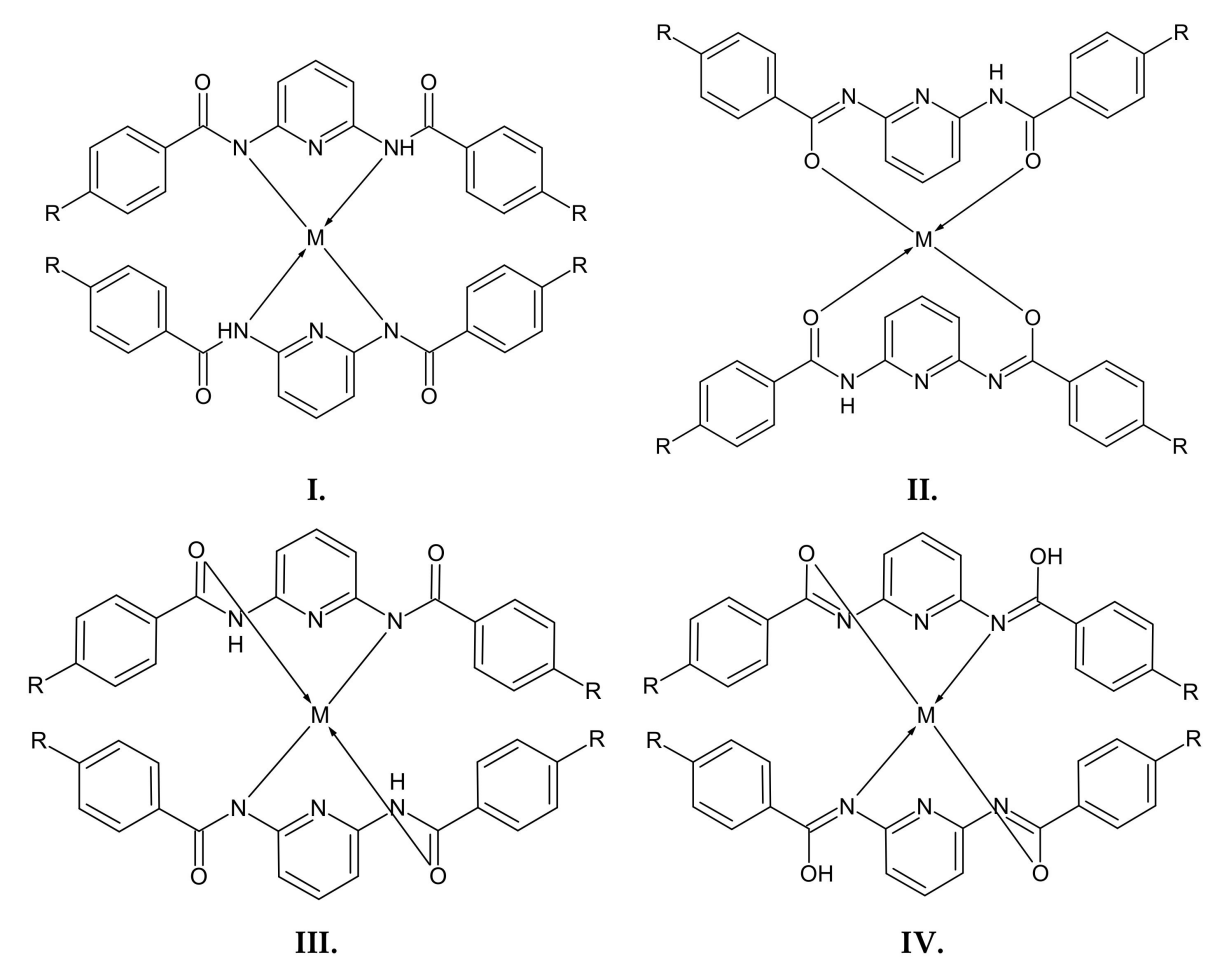
The proposed structures (I, II, III, and IV) of the complexes of compounds A1–A3 and metal ions (M(II)).

It has been reported that among the nitrogen-containing heterocycles, pyridine and its derivatives belong to a group of compounds that play an important role in biological, pharmacological, and agricultural applications [24,25,26]. As a tridentate ligand, 2,6-bis(imino)pyridine is often used for the synthesis of organometallic compounds [27]. Therefore, we carried out a reaction between 2,6-diaminopyridine and the appropriate benzoyl chloride derivative or ester derivative to obtain 2,6-bis(benzoylamino)pyridines substituted in the aromatic ring. 2,6-Diaminopyridine can be an electron donor or proton acceptor [26], so these derivatives can easily form complexes with metal ions. In one of the ways in which to form complexes, there is a possibility that both nitrogen atoms—from the amide groups and the pyridine ring—can be involved in the complexation, which results from the presence of two functional groups of subjected compounds [28]. However, when examining the complex-forming properties of 2,6-diaminopyridine for the separation of d-electron metal ions [26,29], it was found that only the amide group nitrogen atoms (one atom after deprotonation) are involved in the coordination of metal ions, and the pyridine nitrogen atom does not take part in this process. Based on the literature, the following structures between a metal ion and investigated compounds (Figure 1) can be proposed [30]:

Understanding the complexing properties of the newly-synthesized chemical compounds, which are A1–A3 compounds, will make it possible to use them in separation processes such as solvent extraction, sorption, membrane extraction, and transport across polymer inclusion membranes. It is worth highlighting that the currently described compounds are amides, thus are considered as more polar than Schiff bases.

In the case of all of the above-mentioned processes, the key aspect is the correct selection of carriers, which should feature the appropriate complex-forming properties. In parallel, the simple, efficient, and cheap synthesis is beneficial as well as the easy confirmation of molecular structure (NMR, HRMS). However, it should be emphasized that the effectiveness of the recovery of metal ions from aqueous solutions with the use of new non-commercial chemical compounds is also greatly influenced by the proper selection of the experimental conditions.

In this paper, the results of the application of new organic ligands A1–A3 as extractants in solvent extraction and as carriers in membrane extraction for the recovery of copper(II), nickel(II), cobalt(II), and zinc(II) ions from aqueous solutions are described.

## 2. Materials and Methods

### 2.1. Synthesis of 2,6-Bis((benzoyl-R)amino)pyridine (R = H, 4-Me, and 4-NMe_2_) Derivatives

**Scheme 1:**
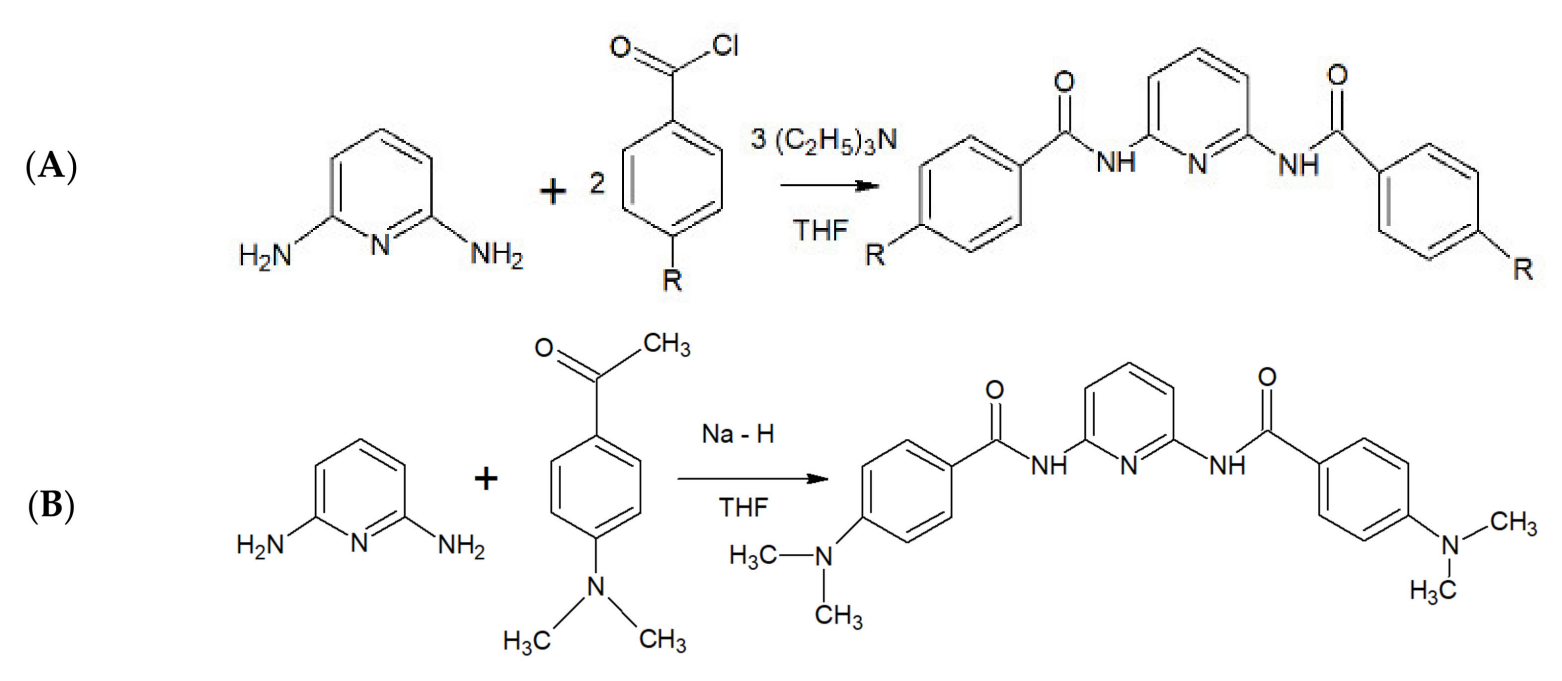
The scheme of the synthesis of A1–A3 compounds during a reaction with (**A**) benzoyl chloride derivatives (R = H, 4-Me) and (**B**) ethyl 4-(dimethylamino)benzoate.

The investigated A1–A3 derivatives were obtained using the reactions presented in Scheme 1.

The steps of the benzoyl chloride derivative synthesis (Scheme 1A) are described below:

I. 2,6-Diaminopyridine (Sigma Aldrich, Poznan, Poland) and triethylamine (Sigma-Aldrich, Poznan, Poland) solutions were magnetically stirred in dry tetrahydrofuran (THF) (Avantor, Gliwice, Poland) (at 0 °C) and then the appropriate amount of 4-R-benzoyl chloride (solution in dry THF) was added dropwise to the mixture over a 60 min period. The 4-R-benzoyl chloride derivatives utilized included benzoyl chloride and p-toluoyl chloride (Sigma Aldrich, Poznan, Poland).
II. The mixture was stirred overnight. It was heated for 1 h, evaporated, and treated by a saturated water solution of NaHCO_3_, and stirred for 15 min.
III. The obtained solids were re-crystallized twice from ethanol (Sigma Aldrich, Poznan, Poland).

The synthesis reaction with ethyl 4-(dimethylamino)benzoate (Scheme 1B) involved the following steps:

I. 2,6-Diaminopyridine (Sigma Aldrich, Poznan, Poland) and sodium hydride (Sigma Aldrich, Poznan, Poland) solutions were magnetically stirred under a nitrogen atmosphere for 1 h (at 25 °C).
II. The mixture was heated for 1 h and then cooled to r.t. Ethyl 4-(dimethylamino) benzoate in dry tetrahydrofuran was added dropwise to the cooled mixture over a 60 min period.
III. The mixture was heated to boiling point and stirred overnight.
IV. An NH_4_Cl solution (Sigma Aldrich, Poznan, Poland) was added to the cooled mixture and was stirred to allow ammonia to evaporate.
V. The mixture was evaporated under reduced pressure and a obtained solid was crystallized from ethanol (Sigma Aldrich, Poznan, Poland).

The specific descriptions of the syntheses of all investigated compounds as well as their ^1^H NMR and ^13^C NMR spectra obtained using a Bruker Avance III 400 MHz (with a DMSO-d_6_ solution in the case of all compounds synthesized) are provided in the Supplementary Materials, whereas their structures are presented in Table 1.

### 2.2. Mass Spectrometry Experiments

A high-resolution mass spectrometry (HRMS) analysis of compounds A1–A3 was performed using a QExactive Orbitrap™ mass spectrometer (Thermo Fisher Scientific, Bremen, Germany) equipped with a TriVersa NanoMate robotic nanoflow ESI ion source (Advion BioSciences Ltd., Ithaca, NY, USA). Samples of A1–A3 were dissolved in methanol (Avantor, Gliwice, Poland) to achieve a concentration of 1 mmol/dm^3^ before being introduced into the mass spectrometer using an electrospray source. All of the HRMS data were acquired in a positive ion mode within the m/z range of 50–750 and at the resolution of 140,000 (m/z 200). Tandem mass spectrometry experiments (MS/MS) were performed using the higher-energy collisional dissociation mode (HCD) with the normalized collision energy set individually for each compound (in a range of 15–45 eV). The Thermo Xcalibur software (ver. 4.1.31.9) was used to process the obtained HRMS and MS/MS spectra.

### 2.3. Complexation Properties of 2,6-Bis(R-benzoyl-amino)pyridine (R = H, 4-Me, and 4-NMe_2_) Derivatives

The absorption spectra of the complexes of metal ions and the examined compounds were recorded to calculate their stability constants. For this purpose, stock solutions of each salt of metal and methanol solutions of each 2,6-bis((benzoyl-R)amino)pyridines derivatives were made. Then, the appropriate amounts of the salt solution and the methanol solution of the examined compound were mixed to prepare samples for spectrophotometric analysis. The concentration of the ligand and metal ions in the samples was selected in such a way that the absorbance of the start solutions did not exceed one and the concentration ratios of ligand to metal in each of the next solutions were appropriate. The absorption spectra of the samples were recorded with varying molar ratios of the components (ligand:metal). The spectra were recorded in the 200–450 nm wavelength range.

### 2.4. Separation Procedure

#### 2.4.1. Classic Solvent Extraction

The separation of copper(II), nickel(II), cobalt(II), and zinc(II) ions by solvent extraction was conducted. All experiments were performed at 25 ± 0.2 °C. The concentration of metal ions in the aqueous solution was obtained after the appropriate dissolution of the reference metal. The organic solution contained organic ligands A1–A3 (L), which was dissolved in chloroform. The concentration ratio of metal ions in aqueous solution to ligands in organic solution was 1:1, 1:2, and 1:5, respectively. The chloroform solution of the appropriate ligand was added to the same volume of aqueous solution. The volume of both phases (aqueous phase and organic phase) was 2500 µL. The prepared samples were then shaken for one hour. The equilibrium was established after approximately 15, 45, and 60 min by visual observation. It was then checked to see if any changes in the phase volumes had occurred, then the phases were separated and the pH of the aqueous phase was measured. The metal ion concentration in the aqueous phases was determined by atomic absorption spectrophotometry (AAS 240FS Spectrometer, Agilent, Santa Clara, CA, USA). The parameters of classic solvent extraction with 2,6-bis((benzoyl-R)amino)pyridine (R = H, 4-Me, and 4-NMe_2_) derivatives as extractants are shown in Table 2.

#### 2.4.2. The Membrane Extraction Process

##### The Preparation of Polymer Inclusion Membrane

A solution containing 60 wt.% of polyvinylchloride (PVC) as a support, 20 wt.% of a bis(2-ethylhexyl)adipate (ADO) as a plasticizer, and 20 wt.% of an appropriate 2,6-bis((benzoyl-R)amino)pyridine (R = H, 4-Me, and 4-NMe_2_) derivative as an ion carrier in 10 µL tetrahydrofuran was prepared. The membrane was obtained by pouring the received solution on a glass ring. After a slow evaporation of the solvent for 12 h, the resulting polymer inclusion membrane (PIM) was peeled off from the glass plate. Through the next 12 h, PIM was immersed in distilled water. The membranes were homogeneous, transparent, flexible, and had good strength. Mean thickness of membranes was determined in the same way, as described previously [31]. The thickness of the membranes, which were used for transport of copper(II), nickel(II), cobalt(II), and zinc(II) ions, was approx. 0.25 mm.

##### Membrane Extraction Experiments

The circular polymer membranes were immersed in beakers containing metal ions such a copper(II), nickel(II), cobalt(II), and zinc(II). The ratio of concentrations of metal ions in aqueous solutions and appropriate ligands in organic solutions was 1:5. For each examined solution, an appropriate amount of ammonia solution was added to adjust the pH. The pH was above 8. Then, samples were taken from the aqueous phases at regular intervals over the course of 24 h of the conducted experiments. The parameters of membrane extraction processes with A1–A3 compounds as carriers are shown in Table 3.

## 3. Results and discussion

### 3.1. Confirmation of the Structures of 2,6-Bis((benzoyl-R)amino)pyridine (R = H, 4-Me, and 4-NMe_2_) Derivatives Using HRMS and HCD MS/MS Methods

The electrospray ionization high-resolution mass spectrometry method (ESI-HRMS) was used to confirm the molecular masses and formulas of the A1–A3 compounds analyzed, while the HCD MS/MS tandem mass spectrometry method was used to verify the compound structures. As a soft ionization technique, ESI enables most small molecules such as the ones analyzed to be transferred directly from the solvent into the mass spectrometer without a major change in their structures [32]. The HRMS method, characterized by high mass accuracy and sensitivity, enables the precise determination of the elemental composition and charge of the ions formed. The HCD MS/MS technique (called ‘beam-type’ CID) is based on a controlled decomposition of the selected ions of the species examined into smaller fragments, followed by an analysis of the resulting dissociation products, which provides detailed structural information [33,34].

**Figure 2:**
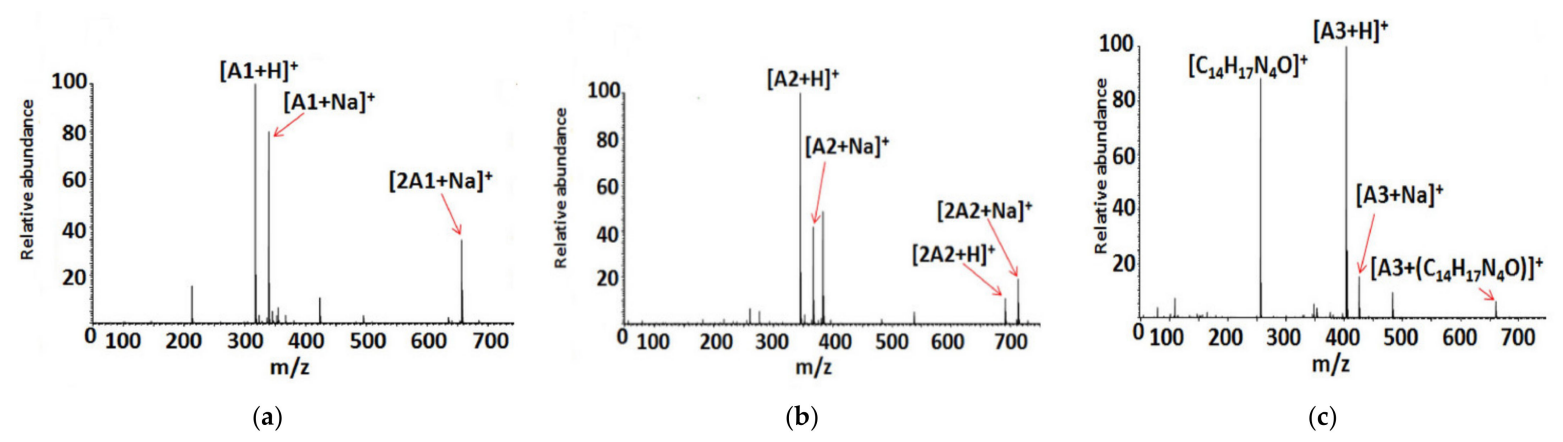
Electrospray ionization (ESI) (+) HRMS mass spectra of compounds: A1 (**a**), A2 (**b**), and A3 (**c**), dissolved in methanol. The unassigned signals correspond to ions, which are not relevant in this study (i.e., complexes formed by solvent molecules, impurities).

Electrospray ionization high-resolution mass spectrometry of the samples (dissolved in methanol) containing the compounds analyzed resulted in the formation of the following singly charged ions: [M+H]^+^, [M+Na]^+^, and [2M+Na]^+^, where M = A1, A2, and A3. Figure 2a–c shows the ESI HRMS spectra of compounds A1–A3, whereas Table 4 presents the ESI-HRMS data of the main ions generated under the ESI conditions. The formation of sodiated ions, typical for ESI HRMS experiments may be related to the presence of a small amount of Na^+^ in the samples as a result of using certain substrates for the synthesis (i.e., NaHCO_3_) or due to the laboratory glassware utilized [35,36].

The results of the ESI HRMS experiments performed unequivocally confirm that the A1–A3 chemical compounds possess the correct molecular weight and elemental composition. The formation of [C_14_H_17_N_4_O]^+^ ions in the case of the ESI HRMS conducted using a sample containing A3 is most probably related to the decomposition of a small part of the compound molecules in the methanol solution, electrospray ionization process, or the compound storage method. Regardless of their origin, the most intensive signal on the spectrum (Figure 2c) corresponds to singly charged [A3+H]^+^ ions, which confirms that only a small part of A3 molecules was decomposed.

The results of ESI HCD MS/MS experiments performed to confirm the structures of the A1–A3 compounds together with the relevant ESI HCD MS/MS mass spectra obtained for singly charged protonated ions of analysed molecules are presented in Table 5.

The results of the tandem mass spectrometry experiments performed indicate that the main HCD dissociation processes of all 2,6-bis((benzoyl-R)amino)pyridine (R = H, 4-Me, and 4-NMe_2_) derivatives are similar, regardless of the differences in their structure. Some generated products are the result of a simple breaking of molecular bonds (i.e., [C_7_H_5_O]^+^, [C_12_H_9_N_2_O]^+^ in the case of [A1+H]^+^; [C_8_H_7_O]^+^, [C_13_H_11_N_2_O]^+^ for [A2+H]^+^; [C_8_H_12_N]^+^, [C_9_H_10_NO]^+^, and [C_15_H_15_N_4_O_2_]^+^ in the case of [A3+H]^+^), other fragments originate from more complex intramolecular rearrangements, which are typical for HCD MS/MS (i.e., [C_12_H_11_N_2_O_2_]^+^ in the case of [A1+H]^+^; [C_13_H_13_N_2_O_2_]^+^ for [A2+H]^+^, respectively). While a detailed analysis of the fragmentation processes was not the subject of this work, the results of the higher energy collisional dissociation experiments performed for the singly charged protonated ions of the A1–A3 compounds make it possible to confirm the structures of the species analyzed.

### 3.2. Complexation Properties of 2,6-Bis((benzoyl-R)amino)pyridine (R = H, 4-Me, and 4-NMe_2_) Derivatives

The spectrum in the UV–VIS region is called the electronic absorption spectrum, but strictly, it is the electron-oscillation-rotation spectrum. The energy of a molecule is the sum of electron, oscillation, and rotation energies. Since the electronic transition is accompanied by transitions from a specific sequence of oscillatory and rotational sublevels to their respective combinations in the excited state, the spectrum consists of absorption bands, not single lines. In organic compounds, the absorption of radiation in the UV–VIS region leads to changes in the energy states of valence electrons, causing the transfer of an electron from one orbital to another with higher energy.

**Figure 3:**
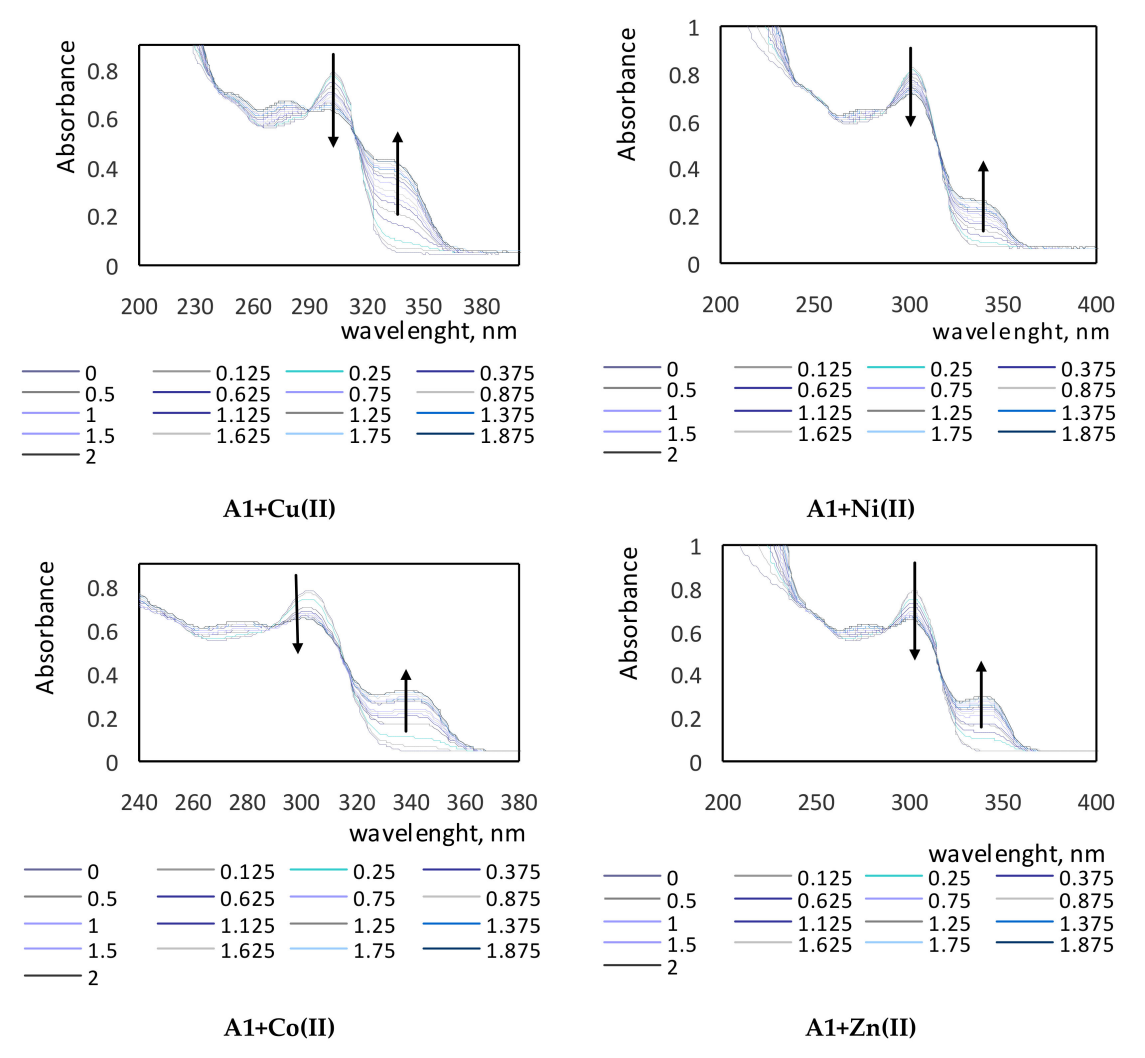

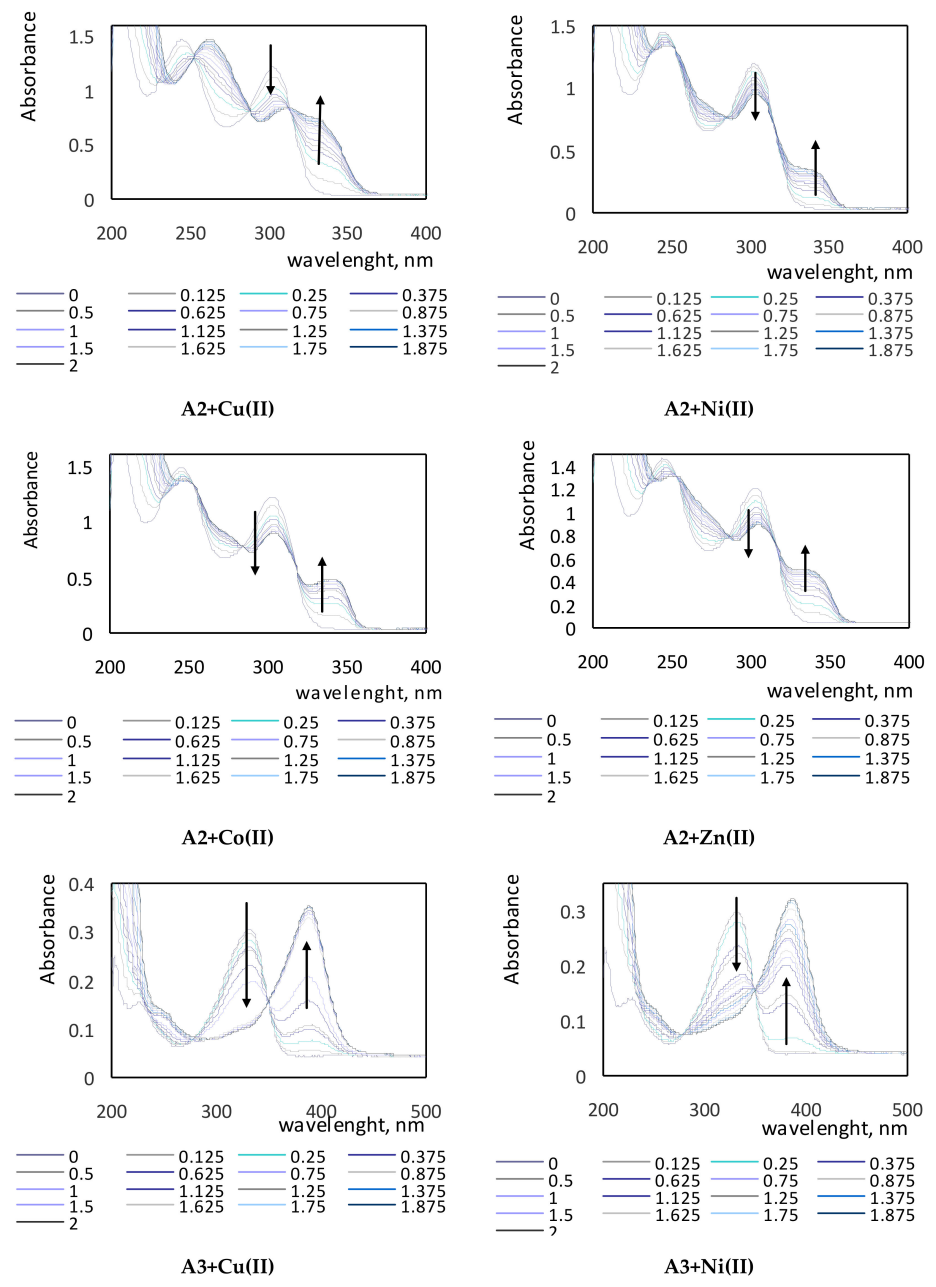

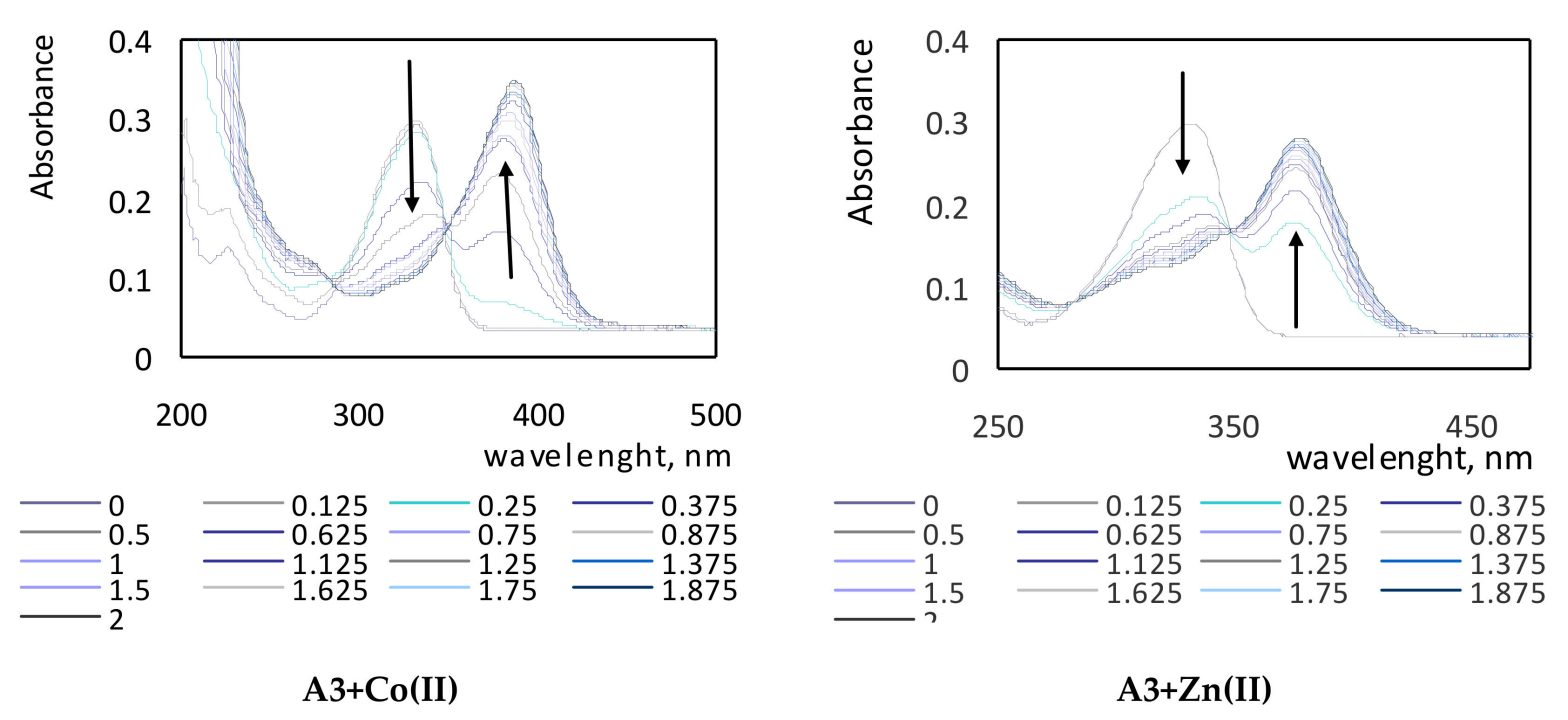
Absorption spectra of complexes of compounds A1–A3 with various metal ions in various molar ratios of ligand to metal.

The absorption spectra of complexes of the tested compounds A1–A3 with various metal ions are shown in Figure 3. On the obtained spectra, the characteristic bands in the UV region are visible. Maxima of absorption for clear A1, A2, and A3 ligands in methanol were observed at wavelengths of 303 nm, 246 nm, and 303 nm (two maxima of absorption), and 333 nm, respectively. On each spectrum, the vanishing band of the ligand and the appearance of new ones corresponding to the complex (ligand + cation) with the isosbestic points were recorded. The shapes of the spectra for each studied compound differed because of the presence of various substituents located in the molecules of investigated derivatives A1–A3. Spectra of the A1 compound showed a hypochromic effect, while in the spectra of the A2 and A3 compounds, bathochromic shifts were visible. The bathochromic shifts are clearly shown for the A2+Cu(II) spectrum and for all spectra of the A3 compound.

Based on the recorded absorption spectra, the stability constants (K) of the created complexes were calculated using the known method [37].

The determined values of the stability constants (log K) of complexes are shown in Table 6. The values of log K_1_, log K_2_ were assigned to stability constants of complexes, in which the molar ratio of ligand:metal ions was 1:1 and 1:2, respectively.

The higher value of the stability constant (log K > 1.0) indicates that at equilibrium, the activity of the complex is larger than the product of activities of the metal ion and ligand (Equation (1)).

mL + [Me(H_2_O)_m_]^n+^ ⇄ [MeL_n_]^n+^ + mH_2_O (1)

As a result, the high value of log K indicates that the ligand binds to the metal ion more strongly than water molecules. The stability constant is then used as a measure of the thermodynamic stability of the complex. An increase in the number of ligands in the coordination sphere usually leads to a decrease in the number of water molecules to be replaced. This is why in some cases, it is found that complexes with a larger number of ligands are more stable than others because of unusual structural changes and differences in the electronic configuration of the metal ion. The complexes with a higher crystal field stabilization energy value will be stable, and the stability constant for those complexes will be high [38].

Due to their structure (i.e., the presence of deprotonated NH and C=O groups in their molecules,) the tested derivatives belong to the so-called Lewis bases. Such compounds easily react with metal ions—the so-called Lewis acids. Therefore, in aqueous solutions, complexes of the tested derivatives with selected metal ions are characterized by quite stable structures. They can be compared with the stability of complexes formed by β-diketones, whose log K_1_ for complexes with metal ions such as cobalt and nickel has a value amount to 5.

The stability constants of complexes of a derivative with the –N(CH_3_)_2_ groups with the chosen metal ions were the highest. This compound created the most stable complexes, especially with copper(II) and nickel(II) ions. This may be explained by a substituent effect of the –N(CH_3_)_2_ group in the deprotonated ligand. The electron donating –N(CH_3_)_2_ moiety increased the basic character of the (O–C–N–C–N)—making it more potent to interaction with the Lewis acid.

The results suggest that the analyzed compounds may be useful in the extraction of different metal ions from aqueous solutions, and thus may be applicable as selective carriers in polymer inclusion membranes that may be used to recover metal ions.

### 3.3. Results of Separation Processes

#### 3.3.1. Classic Solvent Extraction

The solvent extraction of copper(II), nickel(II), cobalt(II), and zinc(II) ions was performed with 2,6-bis((benzoyl-R)amino)pyridine (R = H, 4-Me, and 4-NMe_2_) derivatives as extractants. The experiments were carried out for solutions in which the ligands and metal ions concentration ratios were 1:1, 1:2, and 1:5, respectively. The extraction percentage (*%E_M_*) of the metal ions is described by the following equation:

(2) $$\%E_{M}=\frac{D_{M}}{D_{M}+\frac{V_{aq}}{V_{org}}}\cdot 100\%$$

where *D_M_* is the division ratio determined experimentally; *V_aq_* is the volume of the aqueous phase [dm^3^]; *V_org_* is the volume of the organic phase [dm^3^] (*V_aq_* = *V_org_*, so *V_aq_*/*V_org_* = 1).

The division ratio (Equation (3)) is the ratio of the sum of the concentrations of all the substances in the organic phase (Σ[*M*]*_org_*) to the sum of the concentrations of all the substances in the aqueous phase (Σ[*M*]*_aq_*).

(3) $$D_{M}=\frac{\Sigma {\left[M\right]}_{org}}{\Sigma {\left[M\right]}_{aq}}$$

Obtained results were elaborated using a spreadsheet and additionally, the standard deviation was calculated (Table 7).

The obtained division ratios (Table 7) for the analyzed metal complexes with all compounds increased in the following order: Cu(II) < Zn(II) < Ni(II) < Co(II), for the greater part of the studied extraction processes. Co(II) complexes were the best extracted, and the Cu(II) complexes were the worst extracted. In case of the process using compound A1, this sequence changes to Cu(II) < Ni(II) < Zn(II) < Co(II), for the concentration of metal ions in the solution 0.005 mol/dm^3^. While for the compound A2 and the concentration of 0.002 mol/dm^3^, the value of the division ratio was the highest for Zn(II) ions and the lowest for Co(II).

The percent of the extraction of metal ions with all of the investigated extractants is shown in the plots below.

**Figure 4:**
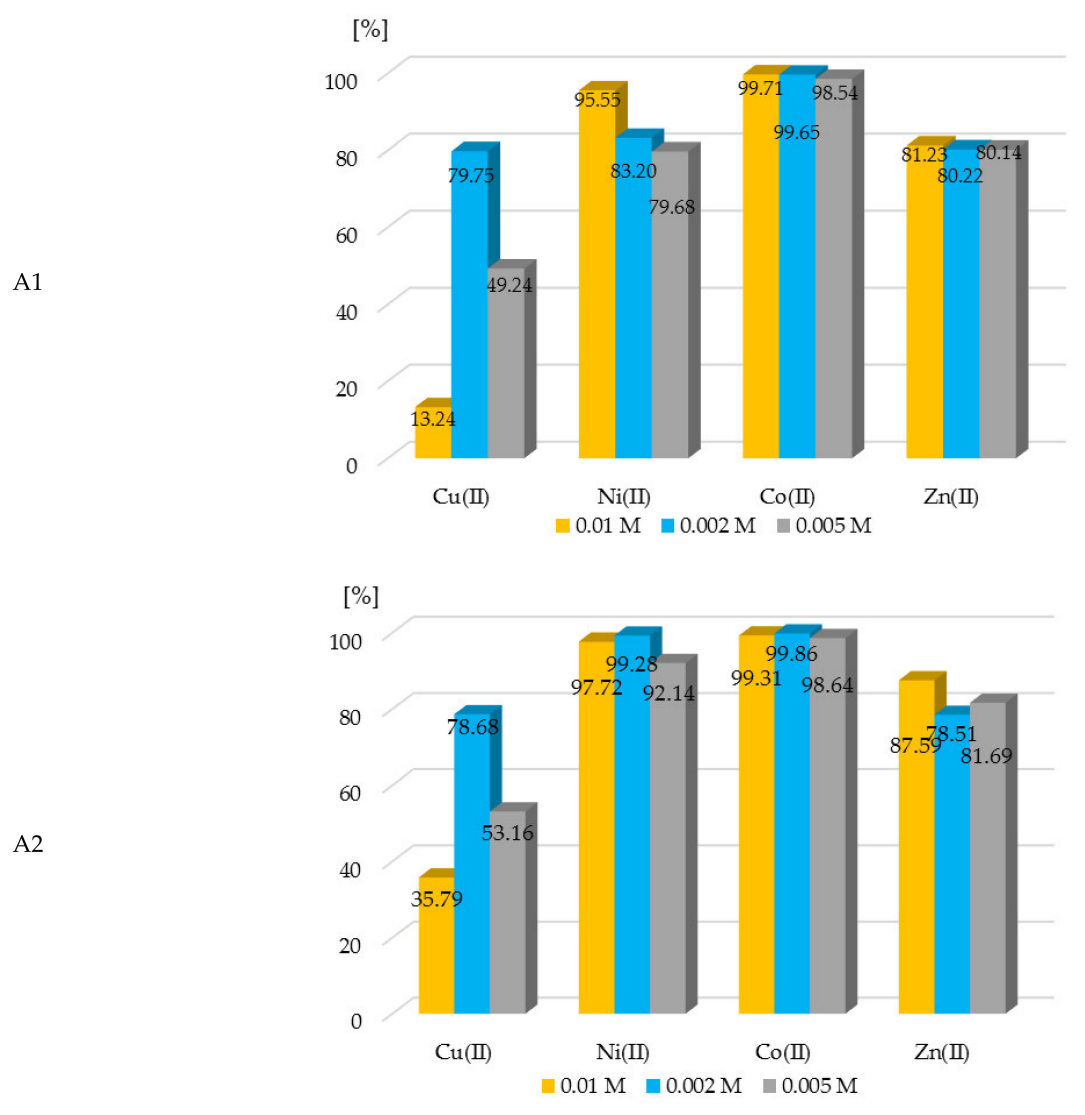

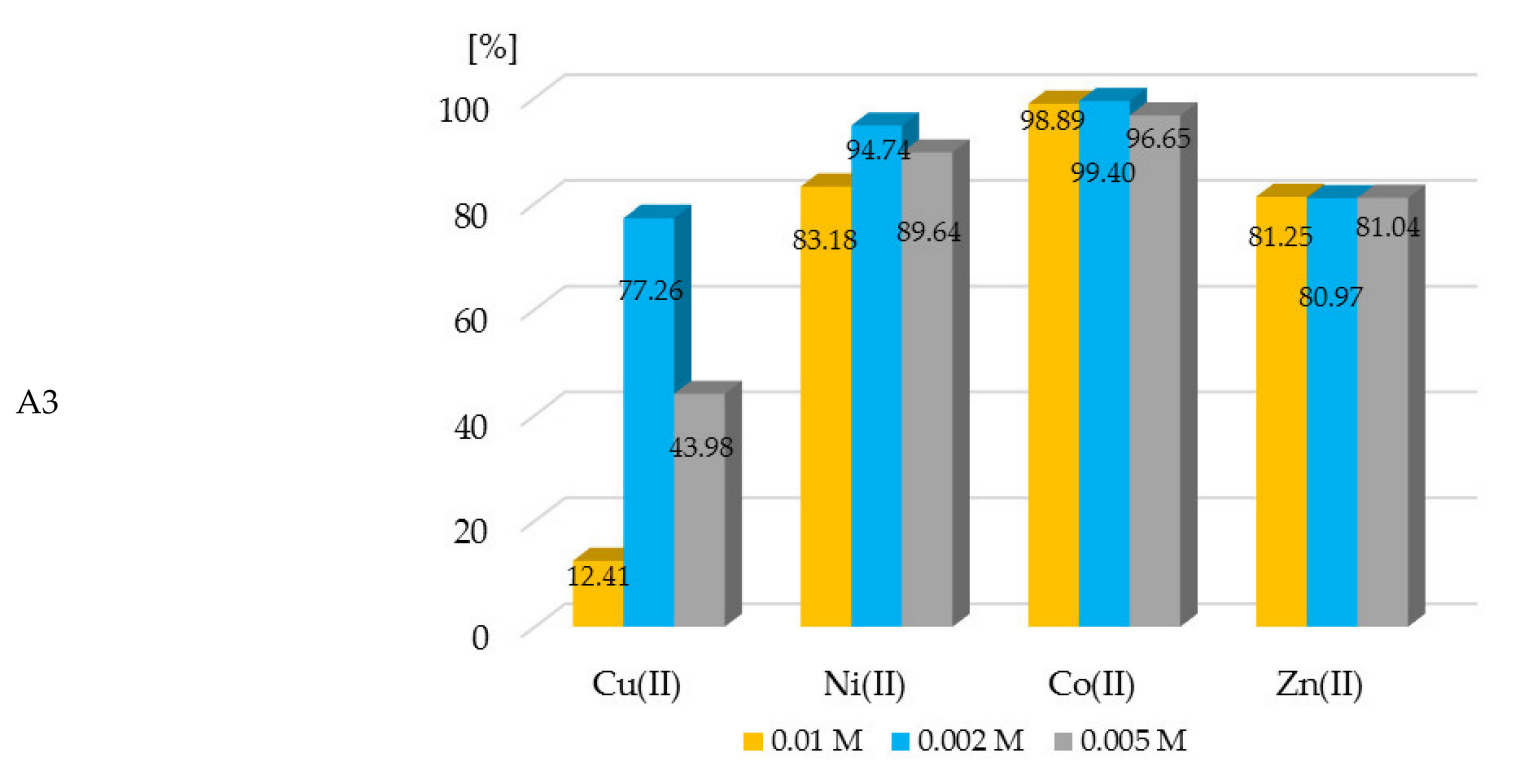
The dependence of the concentration of metal ions in aqueous solution on percent of extraction of metal ions with compounds A1–A3. The given values of the %E_M_ carry ± 0.01%.

The extraction of metal ions with extractants A1–A3 was efficient. The %E_M_ was more than 90% for nickel(II), cobalt(II), and zinc(II) with all A1, A2, and A3 ligands. However, those ligands were not as effective during copper ion extraction (Figure 4). The results of the conducted experiments clearly showed that the proper concentration of metal ions and appropriate ligands had a significant impact on the efficiency of the extraction processes. The conducted spectrophotometric titration (Section 3.2) confirmed the formation of complexes with various M:L ratios including compounds of the type 1:1, 1:2, and 1:5. The extraction studies were performed to take into account the mentioned molar ratios of the metal ion content in the samples to the existing concentration of the extractants. It was found that at the 1:1 molar ratio (M:L), a high recovery of metals was observed, while in most cases with a significant excess of ligand in the solution (1:5), a further slight increase in this recovery was observed.

#### 3.3.2. Membrane Extraction Process

Shortly after the polymer membranes were immersed in a solutions containing Co(II), Ni(II), Co(II), or Zn(II) ions, respectively, the membrane extraction processes were started. Metals ions were adsorbed on the surfaces of the membranes. The ligand molecules (A1, A2, or A3) doped into the polymer membranes bound metals ions thanks to the complexation reactions.

After 24 h of membrane extraction process, the percentage of metal ion removal from the solutions (%*RF*) was also determined (Equation (4)).

(4) $$RF=\frac{c_{0}-c}{c_{0}}\cdot 100\%$$

where *c*_0_ is the initial concentration of metal ions in the feed phase [mol/dm^3^] and *c* is the concentration of metal ions after time t in the feed phase [mol/dm^3^].

**Figure 5:**
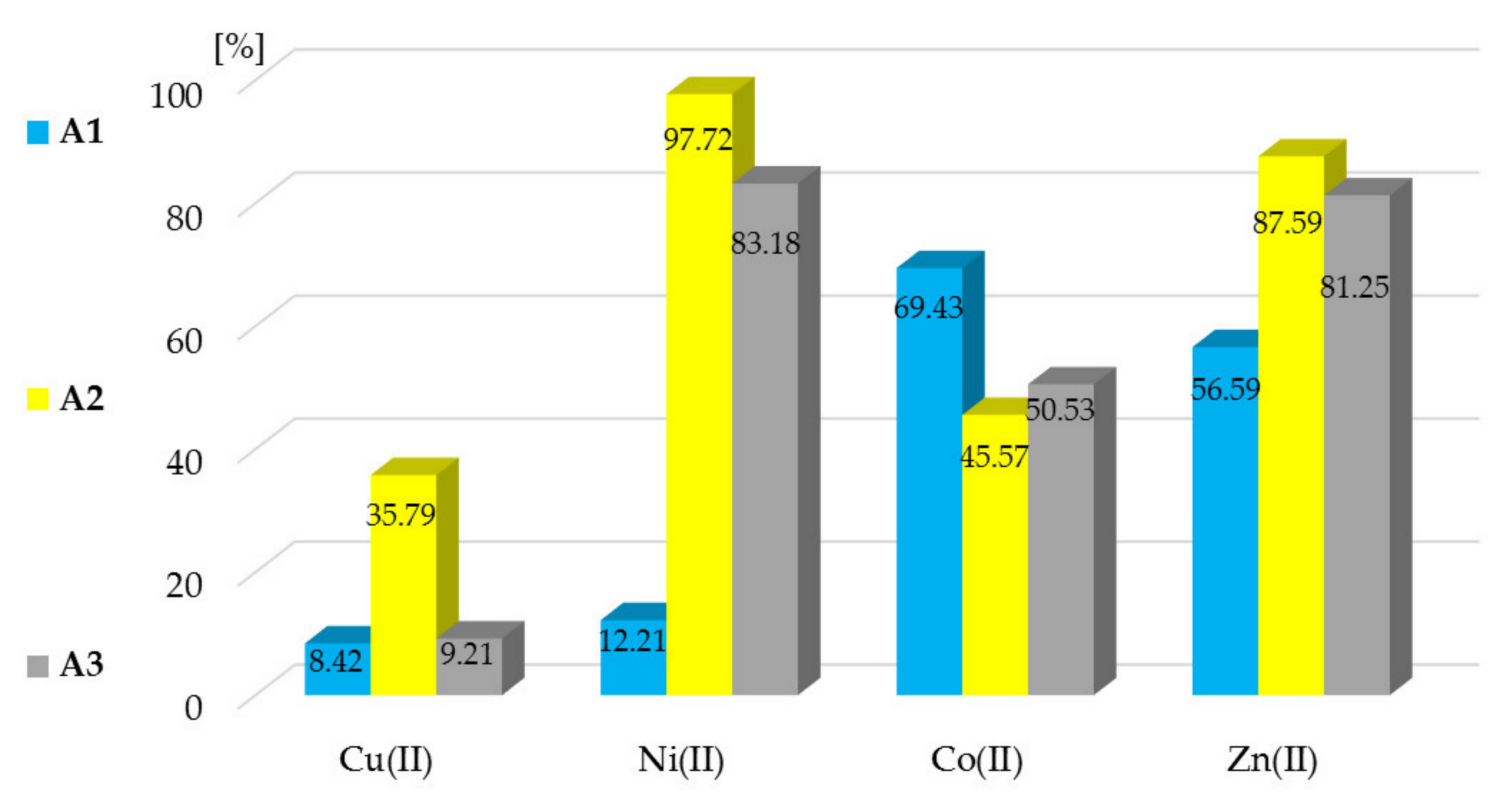
Recovery of metal ions in membrane extraction processes with 2,6-bis((benzoyl-R)amino)pyridine (R = H, 4-Me, and 4-NMe_2_) derivatives as a carriers. The given values of the RF carry ± 0.01%.

The results of the membrane extraction processes performed are presented in Figure 5. Based on these results, it can be unequivocally stated that the efficiency of the membrane processes carried out with the utilization of A1–A3 compounds as carriers was lower than in the extraction processes, in which these compounds were used as extractants. This may be caused by various factors such as, for example, inappropriate selection of the polymer or plasticizer to obtain membranes with the tested compounds. The copper(II) ions recovery was as poor in the case of the membrane process as in classical extraction, regardless of the type of A1–A3 used. As the results obtained for other analyzed ions (Ni(II), Co(II), Zn(II)) were much better (in both types of processes), it can be assumed that the individual properties of metal ions have a significant impact on the extraction and membrane processes. Additionally, for all examined metal ions, the lowest values of metal ions recovery were obtained with the application of compound A1 as a carrier.

The analysis of the metal ion sorption process onto the membranes with 20 wt.% of the investigated compounds used as a carrier was carried out using Equation (5):

(5) $$q_{t}=\left(\frac{c^{i}-c^{t}}{m}\right)\cdot V$$

where *q_t_* is the sorption capacity (mg/g); *V* is the volume of the solution (dm^3^); *m* is the mass of the sorbent (g), and *c^i^* and *c^t^* are the analytical metal ion concentrations in the solution at the beginning and after an appropriate time of the sorption process (mol/dm^3^), respectively.

The sorption capacity of the membrane with all of the ligands A1–A3 as carriers after 24 h of the sorption of investigated metal ions is presented in Table 8.

The highest q_t_ of investigated membranes was obtained for Zn(II) and Co(II) ions sorption and was equal to 3.81 mg/g and 3.42 mg/g, respectively. The lowest q_t_ was observed for Cu(II) ions (0.17 mg/g). The received results can be compared with other literature data (e.g., the sorption capacity of sorbent prepared from mixed local conifer sawdust (SW) and fly ash (FA) was 3.22–3.29 mg/g at 293 K [39], and the sorption capacities of novel nanosorbent (NZVI-DETA-PY) for Co(II), Cu(II), and Zn(II) ions were 2600 μmol/g, 4750 μmol/g, and 5600 μmol/g, respectively [40].

## 4. Conclusions

In this study, the application of a series of new 2,6-bis((benzoyl-R)amino)pyridine (R = H, 4-Me, and 4-NMe_2_) derivatives for the removal of metal ions (copper(II), nickel(II), cobalt(II), and zinc(II)) from aqueous solutions was described.

The compounds A1–A3 were obtained in the condensation reactions of 2,6-diaminopyridine with benzoyl chloride derivatives and with ethyl 4-(dimethylamino)benzoate, respectively. NMR and electrospray ionization high-resolution mass spectrometry (ESI HRMS) and higher energy collisional dissociation tandem mass spectrometry (HCD MS/MS) methods were successfully used to confirm the structures of all synthesized ligands. Given the high mass accuracy of HRMS mass spectrometry and a relatively easy and straightforward interpretation of the simple fragmentation spectra (HCD MS/MS) of the analyzed compounds, there is no doubt about the elemental composition, charge, or structure of the ions formed.

The experiments of the complexation properties confirmed that the series of new compounds exhibited high complex-forming properties. The various molar ratio of ligand:metal was proved to form complex metal ion complexes with the investigated ligands. Application of the spectrometric method allowed us to determine the ratio of ligand to the metal in the studied complexes as well as their stability, which was quite high.

Thanks to the complex-forming properties of the compounds A1–A3, it is possible to use synthesized compounds as very efficient extractants for removing metal ions from aqueous solutions. Studied derivatives can also be used as carriers in the membrane processes. However, to increase the efficiency of membrane processes, further research is needed, where membranes containing a different polymer and/or plasticizer will be used. Since the synthesis of chemical compounds used in this study as extractants and carriers is relatively easy and cheap, and these compounds have strong complexing properties, the results of this research may prove to be a new direction in the search for simple organic ligands to remove metal ions from waste.

## Figures and Tables

**Table 1:** The properties of the A1–A3 compounds obtained.

Structure of compound	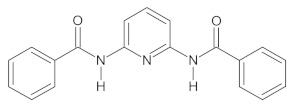
Chemical name	2,6-bis(benzoylamino)pyridine (A1)
Molecular formula	C_19_H_15_N_3_O_2_
Monoisotopic mass [Da]	317.1164
Structure of compound	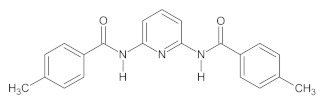
Chemical name	2,6-bis(4-methylbenzoylamino)pyridine (A2)
Molecular formula	C_21_H_19_N_3_O_2_
Monoisotopic mass [Da]	345.1478
Structure of compound	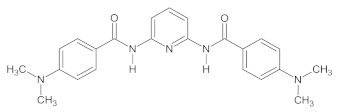
Chemical name	2,6-bis (4-(N,N-dimethylamino)benzoylamino)pyridine (A3)
Molecular formula	C_23_H_25_N_5_O_2_
Monoisotopic mass [Da]	403.2008

**Table 2:** The parameters of classic solvent extraction processes.

Metal Ions	Ligand	M:L	pH	C_[M]_	C_[L]_	T	µ
Cu(II)	A1 C_19_H_15_N_3_O_2_	1:1	10.016	0.010	0.010	20	200
		1:2	8.256	0.005	0.010	22	300
		1:5	10.424	0.002	0.010	25	400
	A2 C_21_H_19_N_3_O_2_	1:1	10.145	0.010	0.010	20	200
		1:2	7.985	0.005	0.010	22	300
		1:5	10.753	0.002	0.010	25	400
	A3 C_23_H_25_N_5_O_2_	1:1	10.160	0.010	0.010	20	200
		1:2	8.157	0.005	0.010	22	300
		1:5	10.792	0.002	0.010	25	400
Ni(II)	A1 C_19_H_15_N_3_O_2_	1:1	10.298	0.010	0.010	20	200
		1:2	7.845	0.005	0.010	22	300
		1:5	10.510	0.002	0.010	25	400
	A2 C_21_H_19_N_3_O_2_	1:1	10.043	0.010	0.010	20	200
		1:2	7.955	0.005	0.010	22	300
		1:5	10.573	0.002	0.010	25	400
	A3 C_23_H_25_N_5_O_2_	1:1	10.134	0.010	0.010	20	200
		1:2	8.254	0.005	0.010	22	300
		1:5	10.580	0.002	0.010	25	400
Co(II)	A1 C_19_H_15_N_3_O_2_	1:1	9.920	0.010	0.010	20	200
		1:2	8.5668	0.005	0.01	22	300
		1:5	10.463	0.002	0.01	25	400
	A2 C_21_H_19_N_3_O_2_	1:1	10.050	0.010	0.01	20	200
		1:2	7.756	0.005	0.01	22	300
		1:5	10.553	0.002	0.01	25	400
	A3 C_23_H_25_N_5_O_2_	1:1	10.139	0.010	0.01	20	200
		1:2	8.651	0.005	0.01	22	300
		1:5	10.645	0.002	0.01	25	400
Zn(II)	A1 C_19_H_15_N_3_O_2_	1:1	10.216	0.010	0.01	20	200
		1:2	8.654	0.005	0.01	22	300
		1:5	10.728	0.002	0.01	25	400
	A2 C_21_H_19_N_3_O_2_	1:1	10.138	0.010	0.01	20	200
		1:2	7.859	0.005	0.01	22	300
		1:5	10.716	0.002	0.01	25	400
	A3 C_23_H_25_N_5_O_2_	1:1	10.209	0.010	0.01	20	200
		1:2	7.6591	0.005	0.01	22	300
		1:5	10.682	0.002	0.01	25	400

where: C_[M]_—the concentration of metal ions [mol/dm^3^], C_[L]_—the concentration of ligands A1–A3 [mol/dm^3^], T—temperature [°C], µ—the mixing speed [mix./minutes].

**Table 3:** The parameters of the membrane extraction processes.

Metal Ions	Ligand	M:L	pH
Cu(II)	A1 C_19_H_15_N_3_O_2_	1:5	9.262
	A2 C_21_H_19_N_3_O_2_	1:5	9.194
	A3 C_23_H_25_N_5_O_2_	1:5	9.428
Ni(II)	A1 C_19_H_15_N_3_O_2_	1:5	10.609
	A2 C_21_H_19_N_3_O_2_	1:5	9.332
	A3 C_23_H_25_N_5_O_2_	1:5	10.895
Co(II)	A1 C_19_H_15_N_3_O_2_	1:5	10.654
	A2 C_21_H_19_N_3_O_2_	1:5	10.456
	A3 C_23_H_25_N_5_O_2_	1:5	9.850
Zn(II)	A1 C_19_H_15_N_3_O_2_	1:5	9.345
	A2 C_21_H_19_N_3_O_2_	1:5	10.850
	A3 C_23_H_25_N_5_O_2_	1:5	9.506

**Table 4:** Electrospray ionization high-resolution mass spectrometry (ESI HRMS) data of the main ions found in the samples containing the A1–A3 compounds analyzed, dissolved in methanol.

Compound A1 (C_19_H_15_N_3_O_2_)		
m/z_meas_	m/z_calc_	Assignment
318.1233	318.1242	[A1+H]^+^
340.1052	340.1062	[A1+Na]^+^
657.2217	657.2226	[2A1+Na]^+^
**Compound A2 (C_21_H_19_N_3_O_2_)**		
**m/z_meas_**	**m/z_calc_**	**Assignment**
346.1538	346.1555	[A2+H]^+^
368.1365	368.1375	[A2+Na]^+^
691.3022	691.3032	[2A2+H]^+^
713.2841	713.2852	[2A2+Na]^+^
**Compound A3 (C_23_H_25_N_5_O_2_)**		
**m/z_meas_**	**m/z_calc_**	**Assignment**
257.1393	257.1402	[C_14_H_17_N_4_O]^+^
404.2077	404.2086	[A3+H]^+^
426.1896	426.1906	[A3+Na]^+^
660.3402	660.3410	[A3+(C_14_H_17_N_4_O)]^+^

**Table 5:** Fragments observed following higher energy collisional dissociation (HCD MS/MS) of the following singly charged protonated ions: [A1+H]^+^, [A2+H]^+^, and [A3+H]^+^, respectively and the relevant ESI HCD MS/MS mass spectra

[A1+H]^+^			ESI HCD MS/MS mass spectrum of [A1+H]^+^
**m/z_meas_**	**m/z_calc_**	**Assignment**	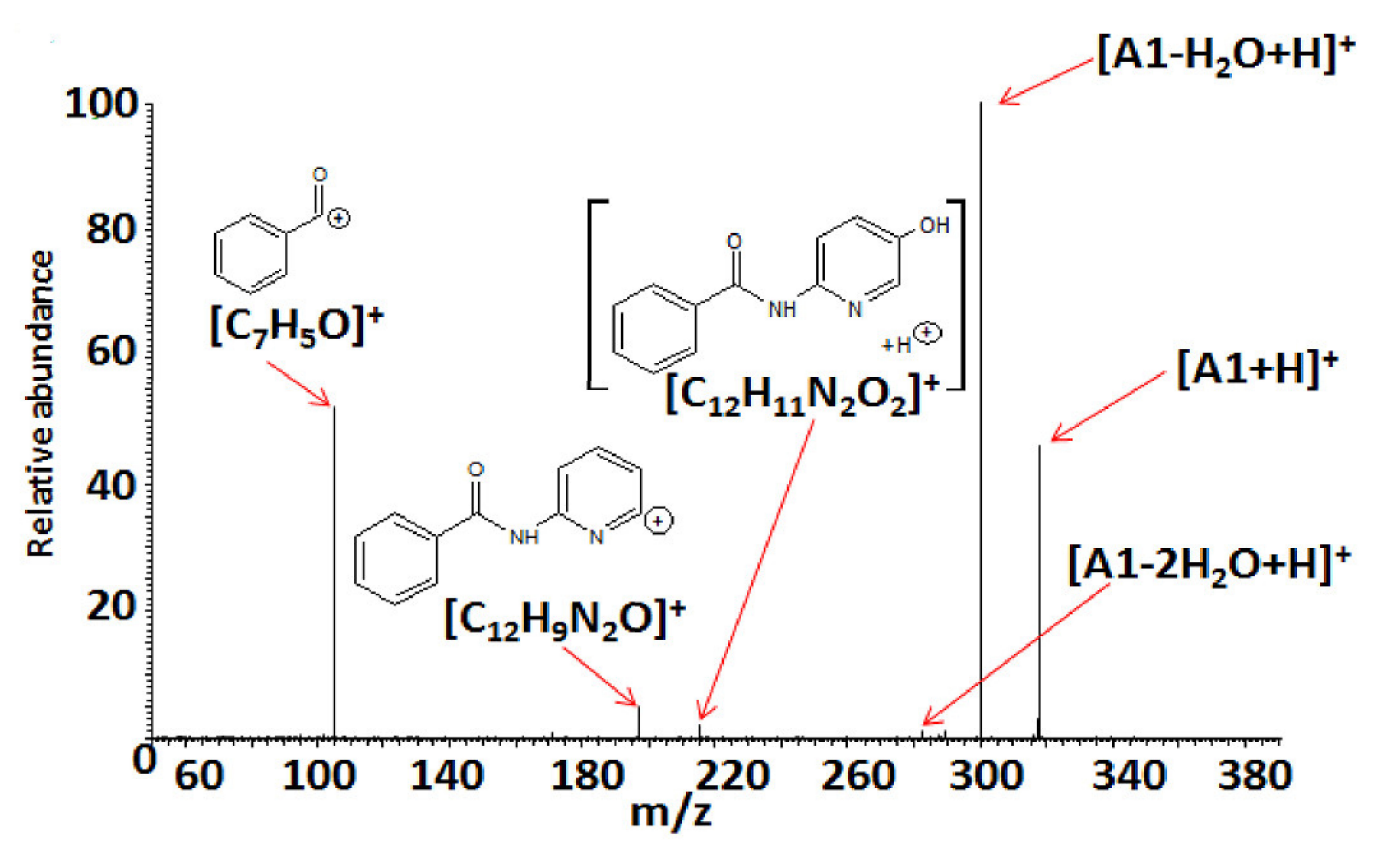
318.1235	318.1242	[A1+H]^+^, (C_19_H_16_N_3_O_2_)	
300.1129	300.1137	[A1-H_2_O+H]^+^, (C_19_H_14_N_3_O_1_)	
282.1032	282.1031	[A1-2H_2_O+H]^+^, (C_19_H_12_N_3_)	
215.0815	215.0820	[C_12_H_11_N_2_O_2_]^+^	
197.0709	197.0715	[C_12_H_9_N_2_O]^+^	
171.0553	171.0558	[C_10_H_7_N_2_O]^+^	
105.0339	105.0340	[C_7_H_5_O]^+^	
95.0496	95.0497	[C_6_H_7_O]^+^	
77.0392	77.0391	[C_6_H_5_]^+^	
**[A2+H]^+^**			**ESI HCD MS/MS mass spectrum of [A2+H]^+^**
**m/z_meas_**	**m/z_calc_**	**Assignment**	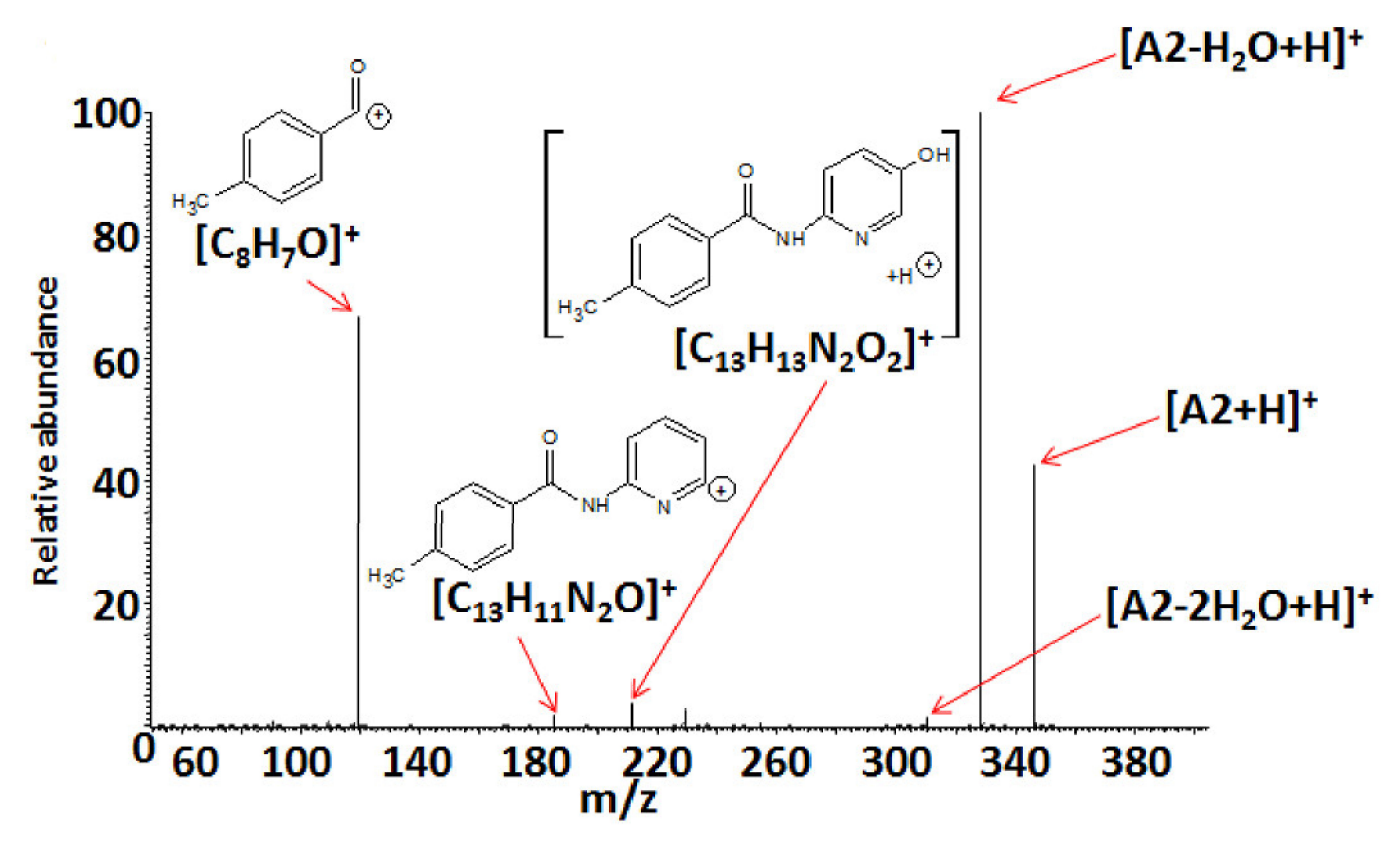
346.1538	346.1555	[A2+H]^+^, (C_21_H_20_N_3_O_2_)	
328.1434	328.1450	[A2-H_2_O+H]^+^, (C_21_H_18_N_3_O_1_)	
310.1330	310.1344	[A2-2H_2_O+H]^+^, (C_21_H_16_N_3_)	
254.0917	254.0929	[C_14_H_12_N_3_O_2_]^+^	
229.0965	229.0977	[C_13_H_13_N_2_O_2_]^+^	
211.0860	211.0871	[C_13_H_11_N_2_O]^+^	
185.0704	185.0715	[C_11_H_9_N_2_O]^+^	
119.0490	119.0497	[C_8_H_7_O]^+^	
109.0649	109.0653	[C_7_H_9_O]^+^	
91.0545	91.0548	[C_7_H_7_]^+^	
**[A3+H]^+^**			**ESI HCD MS/MS mass spectrum of [A3+H]^+^**
**m/z_meas_**	**m/z_calc_**	**Assignment**	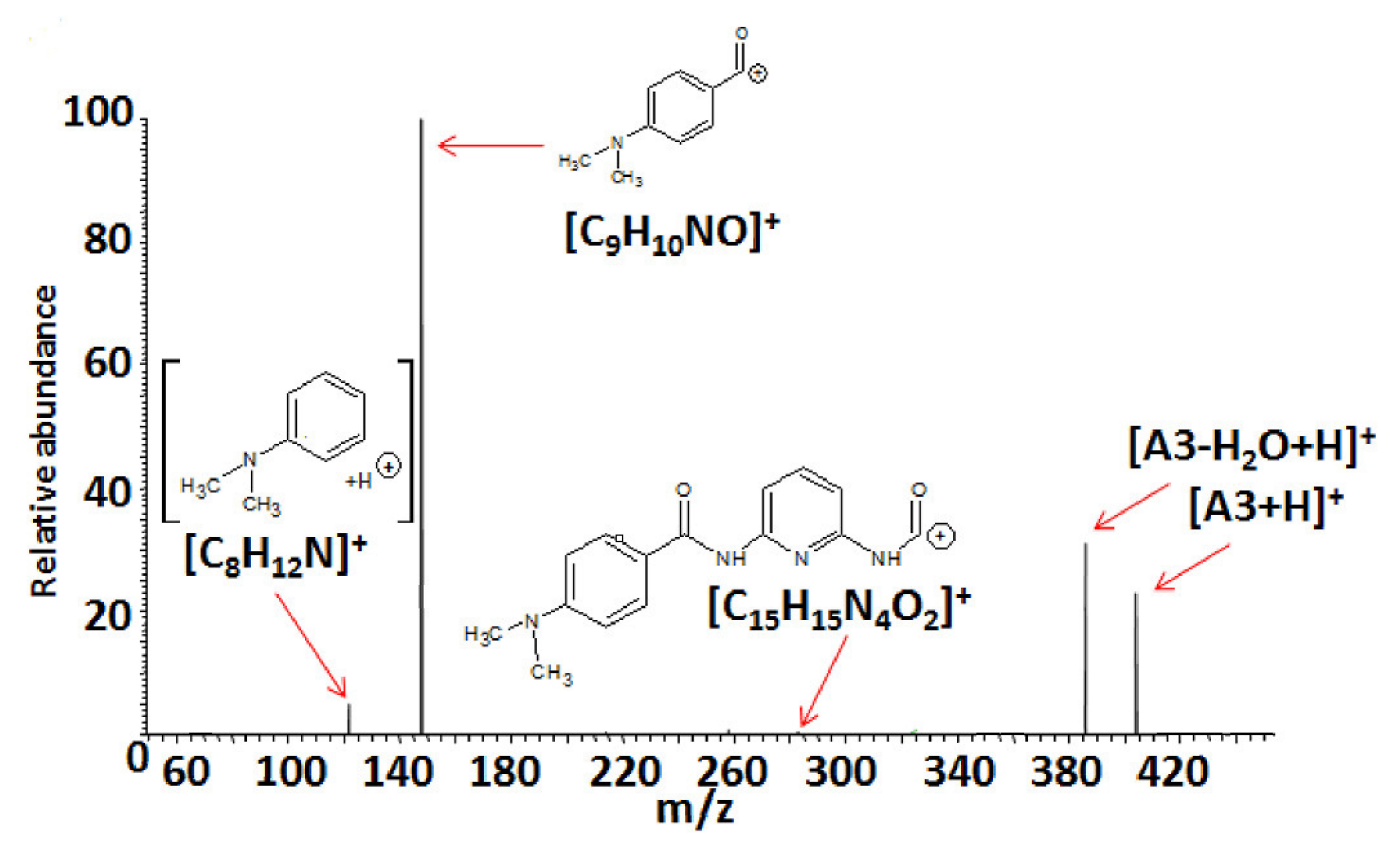
404.2068	404.2086	[A3+H]^+^, (C_23_H_26_N_5_O_2_)	
386.1963	386.1981	[A3-H_2_O+H]^+^, (C_23_H_24_N_5_O_1_)	
356.1463	356.1511	[A3-H_2_O-C_2_H_6_+H]^+^, (C_21_H_18_N_5_O_1_)	
283.1180	283.1195	[C_15_H_15_N_4_O_2_]^+^	
214.0967	214.0980	[C_12_H_12_N_3_O]^+^	
148.0751	148.0762	[C_9_H_10_NO]^+^	
122.0963	122.0970	[C_8_H_12_N]^+^	
79.0547	79.0548	[C_6_H_7_]^+^	

**Table 6:** Stability constants of the complexes of compounds A1–A3 with various metal ions.

Metal Ions	A1—C_19_H_15_N_3_O_2_M = 317.34 g/mol		A2—C_21_H_19_N_3_O_2_M = 345.39 g/mol		A3—C_23_H_25_N_5_O_2_M = 403.47 g/mol	
	log K_1_	log K_2_	log K_1_	log K_2_	log K_1_	log K_2_
Cu(II)	4.398	3.475	4.225	4.724	8.266	9.370
Ni(II)	4.544	4.515	4.500	4.605	7.695	8.131
Co(II)	4.183	4.583	5.809	6.415	4.218	5.836
Zn(II)	3.848	4.942	5.377	5.870	5.908	6.164

The given values of the log K carry ± 0.001.

**Table 7:** The division ratio of metal ions with 2,6-bis((benzoyl-R)amino)pyridine derivatives as extractants.

A1			A2			A3		
Metal Ions	Cm [mol/dm^3^]	D_M_	Metal Ions	Cm [mol/dm^3^]	D_M_	Metal Ions	Cm [mol/dm^3^]	D_M_
Cu(II)	0.01	0.152	Cu(II)	0.01	0.557	Cu(II)	0.01	0.141
	0.002	3.939		0.002	3.689		0.002	3.398
	0.005	0.970		0.005	1.135		0.005	0.785
Ni(II)	0.01	21.486	Ni(II)	0.01	42.765	Ni(II)	0.01	4.944
	0.002	4.953		0.002	137.861		0.002	17.993
	0.005	3.921		0.005	11.722		0.005	8.652
Co(II)	0.01	343.710	Co(II)	0.01	144.544	Co(II)	0.01	88.719
	0.002	281.621		0.002	731.722		0.002	164.552
	0.005	67.493		0.005	73.529		0.005	28.850
Zn(II)	0.01	4.327	Zn(II)	0.01	7.056	Zn(II)	0.01	4.333
	0.002	4.057		0.002	3.653		0.002	4.256
	0.005	4.035		0.005	4.461		0.005	4.274

The given values of the D_M_ carry ± 0.001.

**Table 8:** The sorption capacity of membrane with ligands A1–A3 after 24 h of sorption.

Compound	Metal Ions	q_t_
A1	Cu(II)	0.17
	Ni(II)	0.30
	Co(II)	3.42
	Zn(II)	2.44
A2	Cu(II)	0.79
	Ni(II)	0.92
	Co(II)	2.12
	Zn(II)	3.69
A3	Cu(II)	0.21
	Ni(II)	0.60
	Co(II)	2.07
	Zn(II)	3.81

The given values of the q_t_ carry ± 0.01 mg/g.

## Data Availability

Not applicable.

## Supplementary Materials

The following are available online at https://www.mdpi.com/2077-0375/11/4/233/s1, Figure S1. The ^1^H NMR (A) and ^13^C NMR (B) spectrum of 2,6-(N,N′-dibenzoyl)-diaminopyridine (A1).; Figure S2. The ^1^H NMR (A) and ^13^C NMR (B) spectrum of 2,6-bis(4-methylbenzoyl)-diaminopyridine (A2).; Figure S3. The ^1^H NMR (A) ^13^C NMR (B) spectrum of 2,6-bis(4-dimethylbenzoylamino)pyridine (A3).
