# Supplementary material for: 2,6-Bis((benzoyl-R)amino)pyridine (R = H, 4-Me, and 4-NMe2) Derivatives for the Removal of Cu(II), Ni(II), Co(II), and Zn(II) Ions from Aqueous Solutions in Classic Solvent Extraction and a Membrane Extraction
Source: Membranes (Basel). 2021 Mar 25;11(4):233. doi: 10.3390/membranes11040233 (PMC8064367; doi:10.3390/membranes11040233)
Supplement: Supplementary file 1 [file membranes-11-00233-s001.pdf]

Supplementary materials

The "Supplementary materials" section contains a detailed description of the synthesis of compounds used in the research and their NMR spectra.

2,6-bis(benzoylamino)pyridine (A1). The solution of benzoyl chloride (5.326 g, 50.93 mmol, 2.0 equiv) in dry tetrahydrofuran (50 ml) was added dropwise to an ice-cold suspension of a 2,6-diaminopyridine (2.065 g, 18.92 mmol, 1 equiv) with a triethylamine (7.9 ml, 56.68 mmol, 3 equiv). The reaction mixture was stirred at 20 °C overnight. The solution was evaporated, extracted with chloroform, washed with an aqueous solution of $NaHCO_3$, and the solvent evaporated in vacuo. The solid obtained was twice recrystallised from ethanol. The yield was 39%. ^{1}H NMR (400 MHz, DMSO) (Figure 1.A) δ 10.66 (bs, 2H), 8.00 (d, 4H), 7.92 (m, 3H), 7.62 (t, 2H), 7.54 (m, 4H). ^{13}C NMR (101 MHz, DMSO) (Figure 1.B) δ 166.40, 151.02, 140.52, 134.63, 132.46, 128.96, 128.28, 111.13. ^{15}N NMR (41 MHz, DMSO) 137.5, 264.2. m.p. 179-180 (179-178 [1]).

A

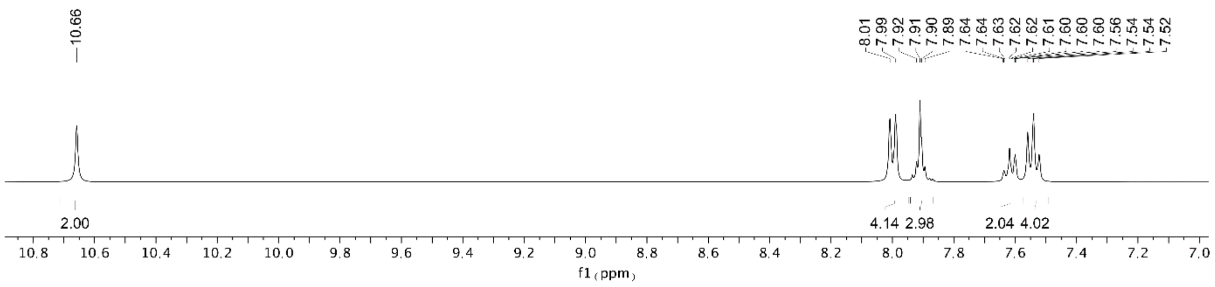

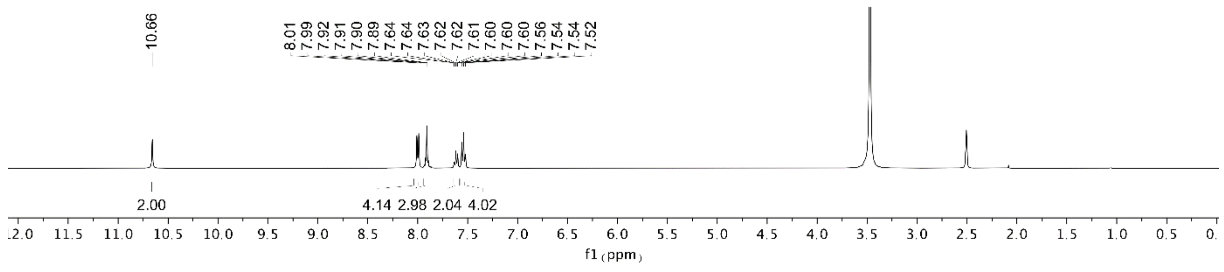

B

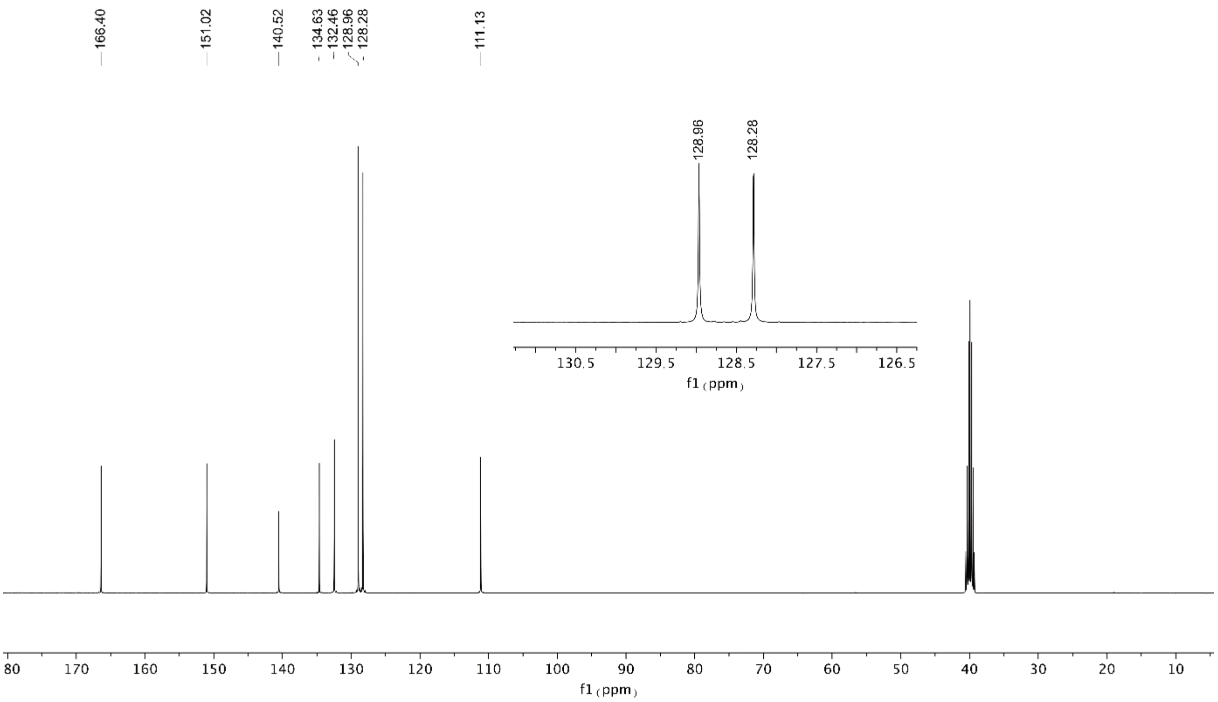

Figure S1. The ^{1}H NMR (A) and ^{13}C NMR (B) spectrum of 2,6-(N,N'-dibenzoyl)-diaminopyridine (A1).

2,6-bis(4-methylbenzoyldiamino)pyridine (A2). The solution of p-toluoyl chloride (5.373g, 37.76 mmol, 2.0 equiv) in dry tetrahydrofuran (50 ml) was added dropwise to an ice-cold suspension of 2,6-diaminopyridine (1.894 g, 17.36 mmol, 1 equiv) with triethylamine (7.2 ml, 51.66 mmol, 3 equiv). The reaction mixture was stirred at 20 °C overnight. The solution was evaporated, extracted with chloroform, washed with an aqueous solution of $NaHCO_3$, and the solvent evaporated in vacuo. The solid obtained was twice recrystallised from ethanol. The yield was 50%. ^{1}H NMR (400 MHz, DMSO) (Figure 2.A) δ 10.52 (bs, 2H), 7.91 (d, 4H), 7.89 (m, 3H), 7.34 (d, 4H). ^{13}C NMR (101 MHz, DMSO) (Figure 2.B) δ 166.17, 151.05, 142.58, 140.45, 131.76, 129.51, 128.31, 110.97, 21.48. ^{15}N NMR (41 MHz, DMSO) 136.7, 263.4. m.p. 215-216 (215-217 [2]).

A

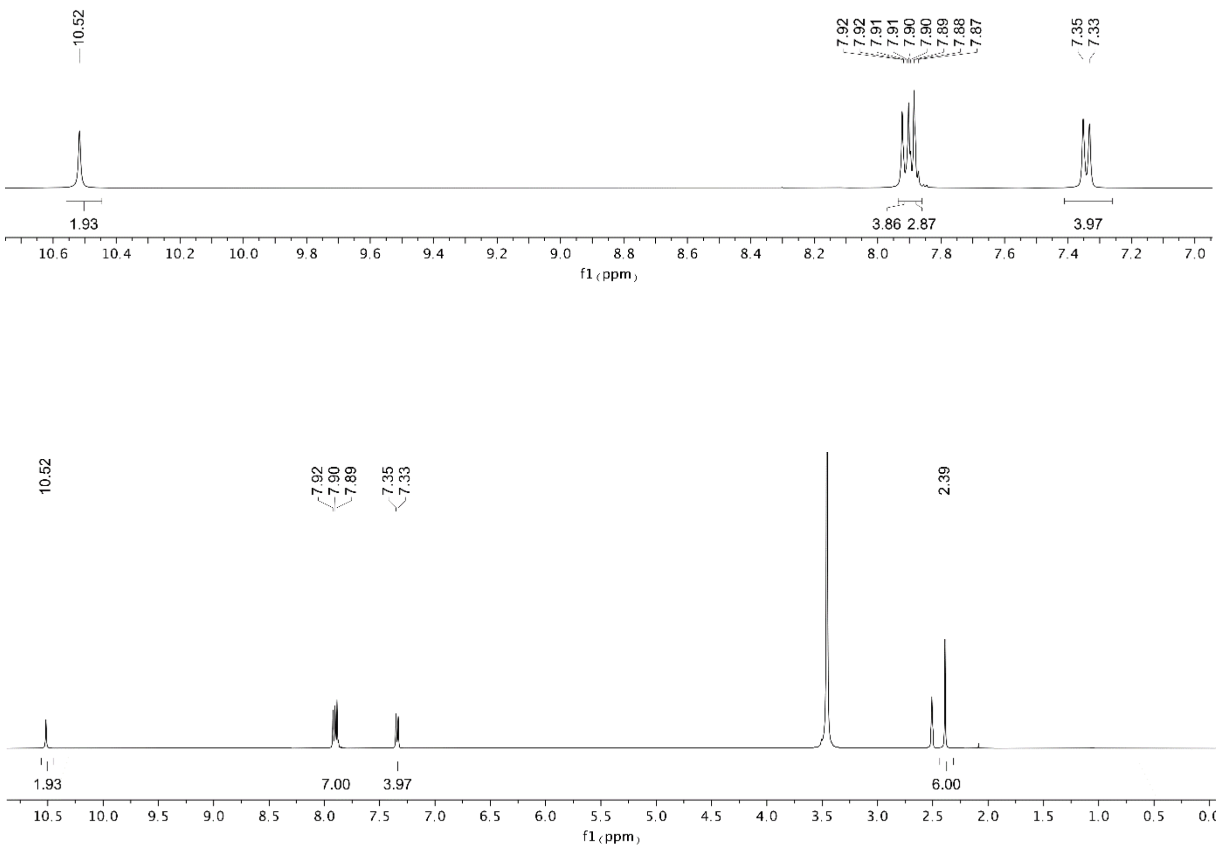

B

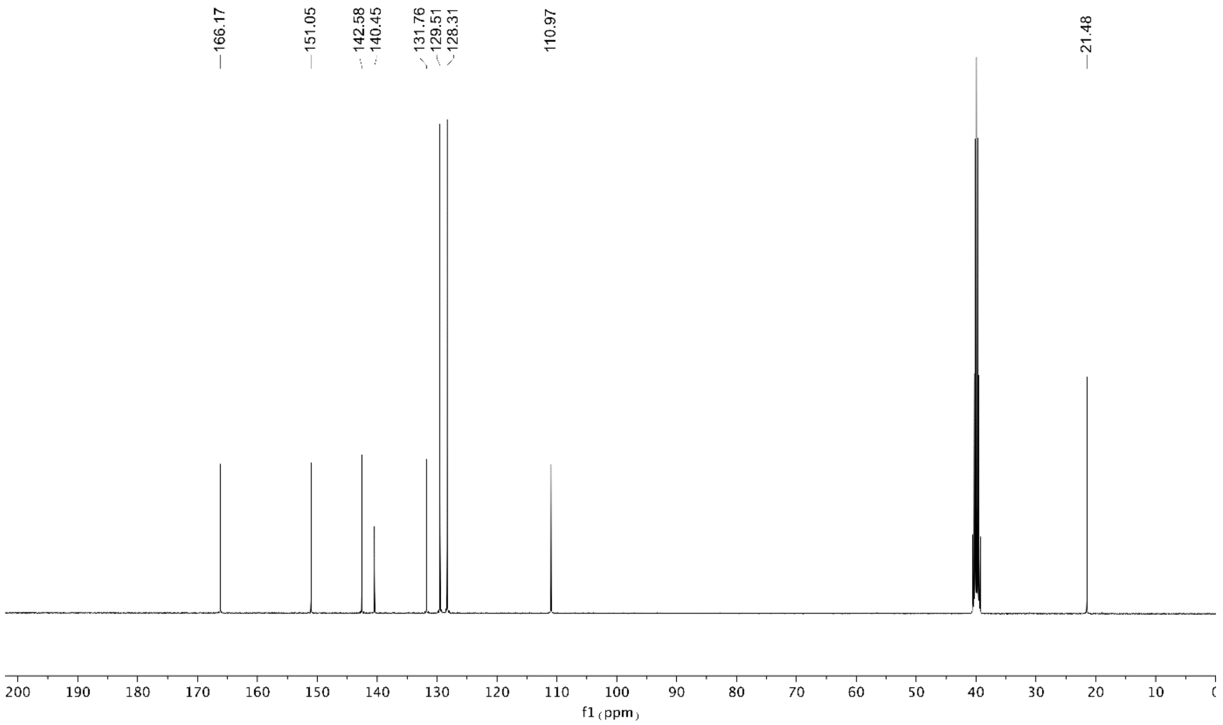

Figure S2. The ^{1}H NMR (A) and ^{13}C NMR (B) spectrum of 2,6-bis(4-methylbenzoyl)-diaminopyridine (A2).

2,6-bis(4-(N,N-dimethylamino)benzoylamino)pyridine (A3). The solution of 2,6-diaminopyridine (2.500 g, 22.90 mmol, 1.0 equiv) in dry tetrahydrofuran (40 ml) was stirred at 25°C under nitrogen atmosphere for 1 hour with sodium hydride (3.6 ml, 60% suspension in oil, 150.01 mmol, 6 equiv). The suspension obtained was stirred at boiling point for 1 hour. Once it cooled down, ethyl-4-(dimethylamino)benzoate (5.748 g, 29.74 mmol, 2 equiv) dissolved in dry THF was added and the solution was warmed up again and the mixture was stirred overnight. Ammonium chloride (8.330 g, 155.72 mmol, 1.7 equiv) solution was added to the cooled solution to quench the reaction. Subsequently, the solvent was evaporated in vacuo and the solid obtained was filtered and twice recrystallised from ethanol. The yield was 30%. ^{1}H NMR (400 MHz, DMSO) (Figure 3.A) δ 10.10 (bs, 2H), 7.91 (d, 4H), 7.86-7.77 (m, 3H), 6.76 (d, 4H), 3.01 (s, 12H). ^{13}C NMR (101 MHz, DMSO) (Figure 3.B) δ 165.77, 153.12, 151.36, 140.14, 129.79, 120.54, 111.32, 110.14. The signal of the -NMe_2 group is overlapped with the solvent. ^{15}N NMR (41 MHz, DMSO) 55.4, 134.4, 261.8. m.p. 221-223 (223.4-226.0 [3]).

A

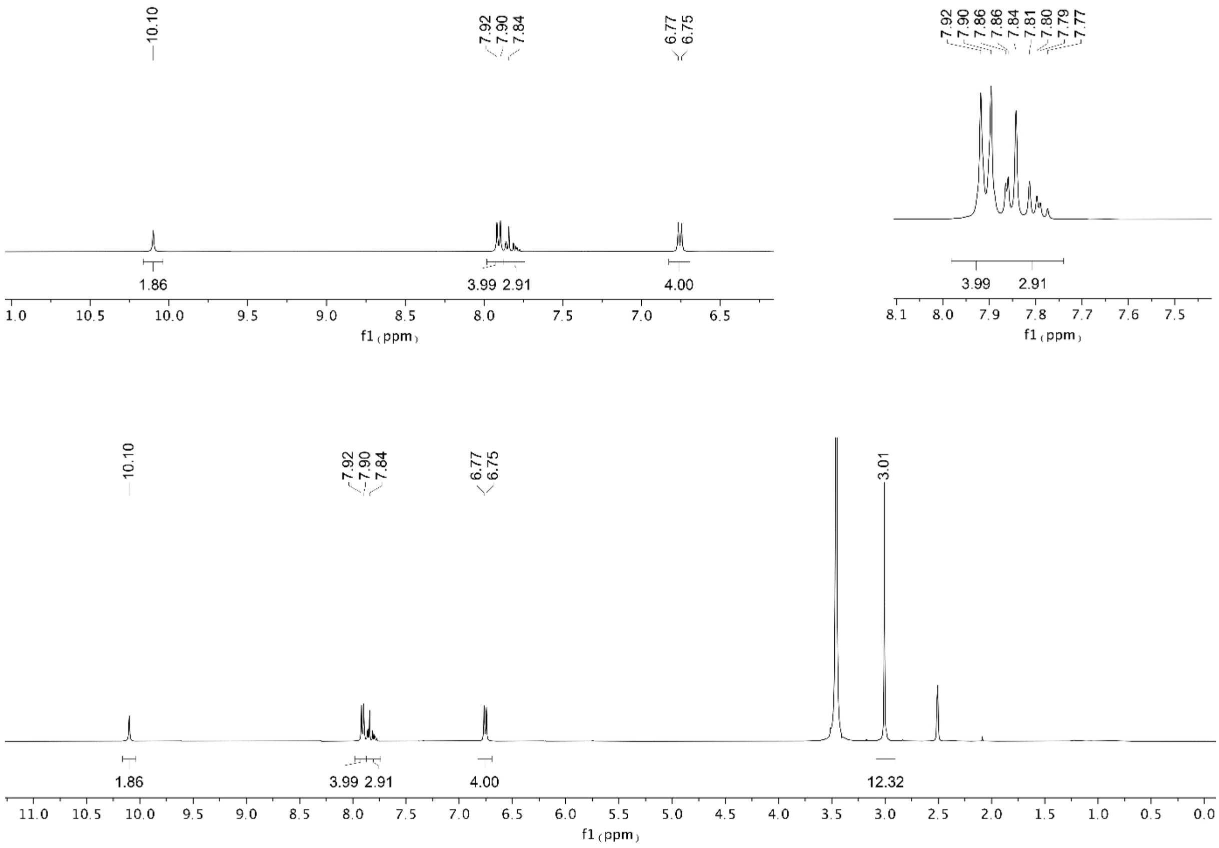

B

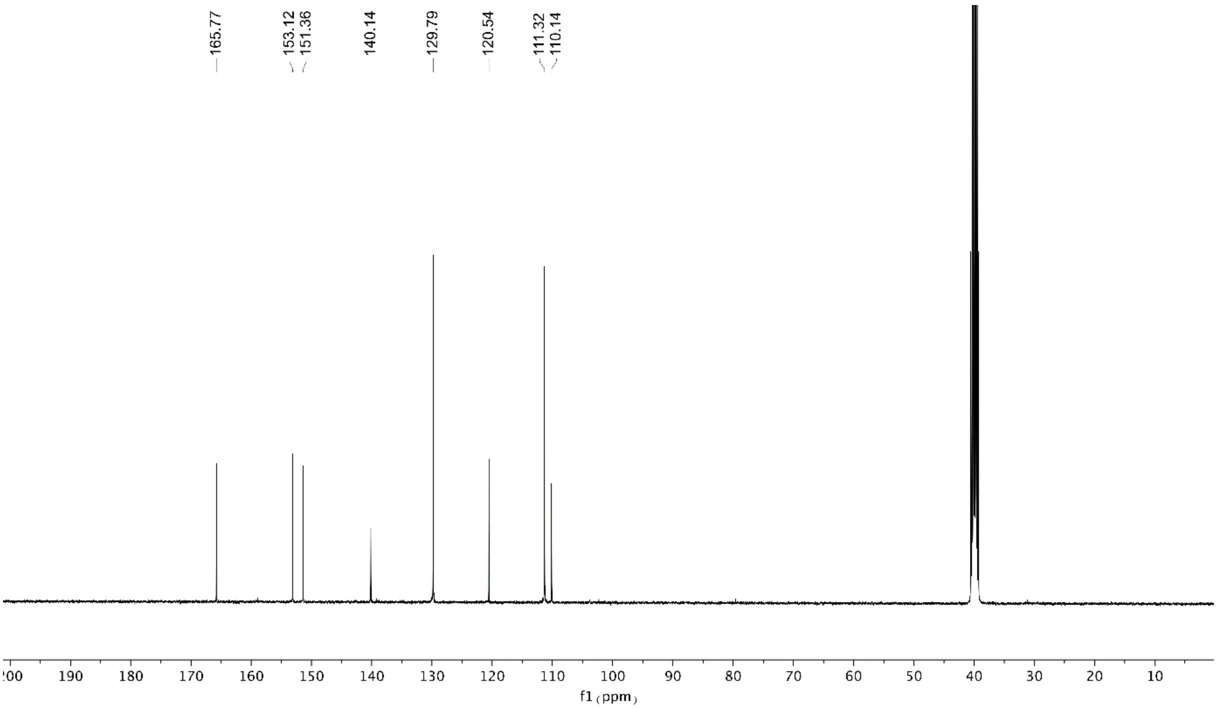

Figure S3. The ^{1}H NMR (A) ^{13}C NMR (B) spectrum of 2,6-bis(4-dimethylbenzoylamino)pyridine (A3).

References

[1] Ruíz-Pérez, K.M.; Quiroz-García, B.; Hernández-Rodríguez, M. Prolinamides of aminouracils, oragnocatalyst modifiable by complementary modules, *Eur. J. Org. Chem.*, **2018**, *41*, 5763-5772. https://doi.org/10.1002/ejoc.201800886.

[2] Langer, P.; Amiri, Sh.; Bodtke, A.; Saleh, N.N.R.; Weisz, K., Görls, H.; Schreiner, P.R. 3,5,7,9-Substituted hexaazaacridines: Toward structures with nearly degenerate singlet-triplet energy separations, *J. Org. Chem.*, **2008**, *73(13)*, 5048-5063. https://doi.org/10.1021/jo8005123.

[3] Grabarz, A.M.; Laurent, A.D.; Jędrzejewska, B.; Zakrzewska, A.; Jacquemin, D.; Ośmiałowski, B. The influence of the π-conjugated spacer on photophysical properties of difluroboranyls derived from amides carrying a donor group, *J. Org. Chem.*, **2016**, *81*, 2280-2292. https://doi.org/10.1021/acs.joc.5b02691.
